# Supplementary material for: Mitochondria serve as a holdout compartment for aggregation-prone proteins hindering efficient degradation
Source: Nat Commun. 2026 May 7;17:4195. doi: 10.1038/s41467-026-72783-0 (PMC13153185; doi:10.1038/s41467-026-72783-0)
Supplement: Supplementary file 1 — Supplementary Information [file 41467_2026_72783_MOESM1_ESM.pdf]

Mitochondria serve as a Holdout Compartment for Aggregation-Prone Proteins hindering Efficient Degradation

Maria E. Gierisch[1,*], Enrica Barchi[1], Mirco Marogna[1], Moritz H. Wallnöfer[1], Maria Ankarcrona[2], Luana Naia[2], Florian A. Salomons[1] and Nico P. Dantuma[1,*]

Supplementary information

The PDF file includes:

Supplementary Figures 1-12

Supplementary Tables 1-2

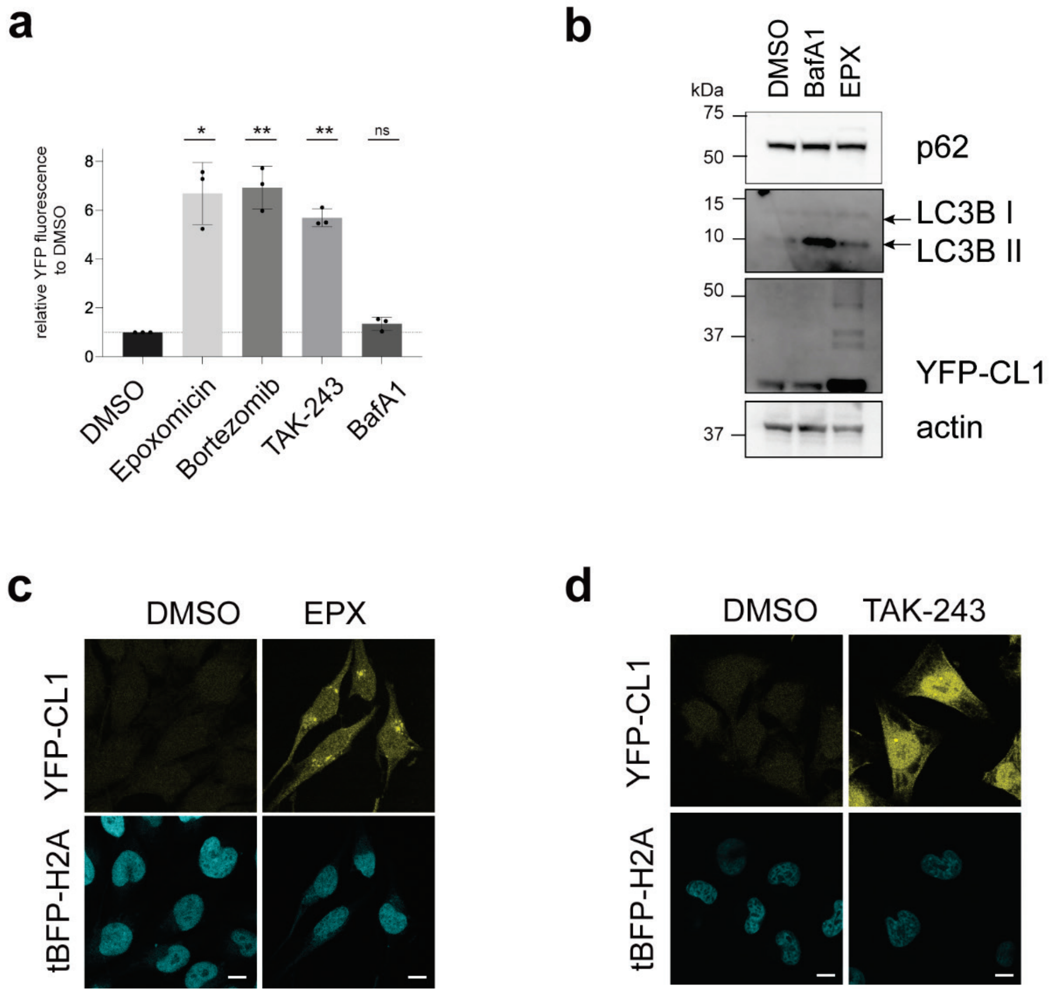

Supplementary Figure 1: (a) MelJuSo YFP-CL1 cells were plated for 72 hours and treated with the proteasome inhibitors epoxomicin (100 nM) and bortezomib (25 nM), the E1 inhibitor TAK-243 (10 μM) or the autophagy inhibitor BafA1 (100 nM) for the last 8 hours and analyzed by flow cytometry (n=3, mean ± SD, one-sample t-test, *P<0.05, **P<0.01, ns: non-significant). **(b)** MelJuSo YFP-CL1 cells were plated for 24 hours and treated with the proteasome inhibitors epoxomicin (100 nM) and the autophagy inhibitor BafA1 (100 nM) for the last 8 hours. Samples were subjected to western blotting with the indicated antibodies. **(c,d)** Stable dual-fluorescent reporter cells were plated on cover slips for 48 hours and treated the last 16 hours with 100 nM epoxomicin (EPX) (c) or the last 6 hours with 1 μM TAK-243 (d) and imaged by confocal microscopy. Representative images, scale bar =10μm.

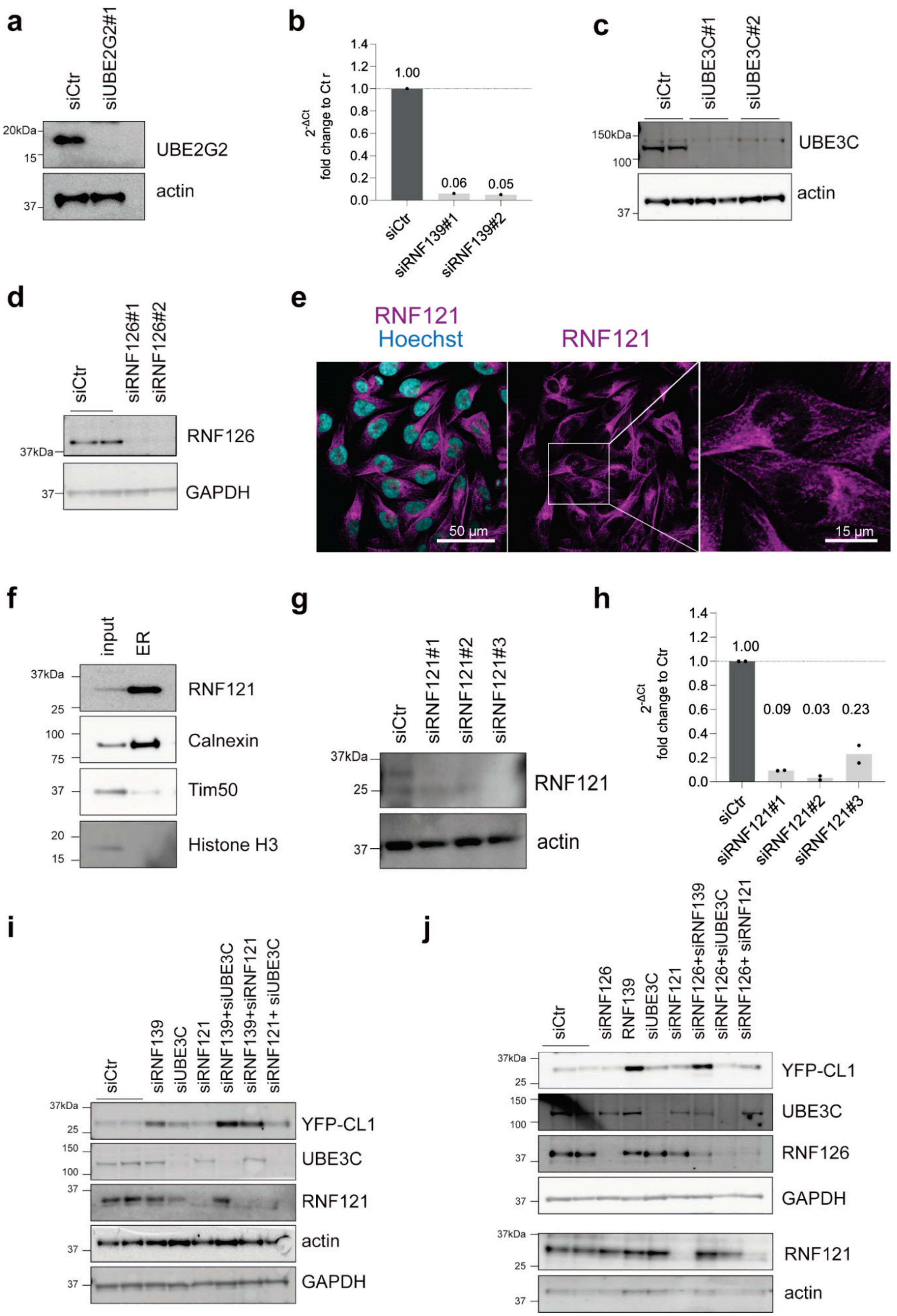
a
siCtr
siUBE2G2#1
20kDa
15
UBE2G2
37
actin
b
1.4
1.2
1.0
0.8
0.6
0.4
0.2
0.0
$2^{-\Delta Ct}$
fold change to Ct r
1.00
0.06
0.05
siCtr
siRNF139#1
siRNF139#2
c
siCtr
siUBE3C#1
siUBE3C#2
150kDa
100
UBE3C
37
actin
d
siCtr
siRNF126#1
siRNF126#2
37kDa
RNF126
37
GAPDH
e
RNF121
Hoechst
RNF121
50 µm
15 µm
f
input
ER
37kDa
25
RNF121
100
75
Calnexin
37
Tim50
20
15
Histone H3
g
siCtr
siRNF121#1
siRNF121#2
siRNF121#3
37kDa
25
RNF121
37
actin
h
1.4
1.2
1.0
0.8
0.6
0.4
0.2
0.0
$2^{-\Delta Ct}$
fold change to Ctr
1.00
0.09
0.03
0.23
siCtr
siRNF121#1
siRNF121#2
siRNF121#3
i
siCtr
siRNF139
siUBE3C
siRNF121
siRNF139+siUBE3C
siRNF139+siRNF121
siRNF121+ siUBE3C
37kDa
25
YFP-CL1
150
100
UBE3C
37
RNF121
37
actin
37
GAPDH
j
siCtr
siRNF126
RNF139
siUBE3C
siRNF121
siRNF126+siRNF139
siRNF126+siUBE3C
siRNF126+ siRNF121
37kDa
25
YFP-CL1
150
100
UBE3C
37
RNF126
37
GAPDH
37kDa
25
RNF121
37
actin

Supplementary Figure 2: (a) MelJuSo YFP-CL1 reporter cells were transiently transfected with an siRNA targeting UBE2G2 for 72 hours and analyzed by western blotting for knockdown efficiency. Relates to flow cytometry data from Fig. 2d. **(b)** MelJuSo YFP-CL1 reporter cells were transiently transfected with siRNAs targeting RNF139 for 72 hours and analyzed by qRT-PCR for knockdown efficiency. Relates to flow cytometry data from Fig. 2d. **(c,d)** MelJuSo YFP-CL1 reporter cells were transiently transfected with an siRNAs targeting UBE3C (c) or RNF126 (d) for 72 hours and analyzed by western blotting for knockdown efficiency. Relates to flow cytometry data from Fig. 2d. **(e)** MelJuSo cells were stained with an anti-RNF121 antibody and imaged by confocal microscopy. Representative image: scale bar = 50 µm, zoom-in image: scale bar = 15 µm. **(f)** MelJuSo cells were fractionated and 10 µg protein of input and ER fraction were analyzed by western blotting using an anti-RNF121 antibody and compartment specific antibodies anti-Tim50, anti-Calnexin and anti-histone H3. **(g,h)** MelJuSo YFP-CL1 reporter cells were transiently transfected with siRNAs targeting RNF121 for 72 hours and analyzed by western blotting (g) and qRT-PCR (h) for knockdown efficiencies. Relates to flow cytometry data from Fig. 2d. **(i,j)** MelJuSo YFP-CL1 reporter cells were transiently transfected with a combination of one or two siRNAs for 72 hours and analyzed by western blotting for knockdown efficiencies with the indicated antibodies. Relates to flow cytometry data from Fig. 2e,f.

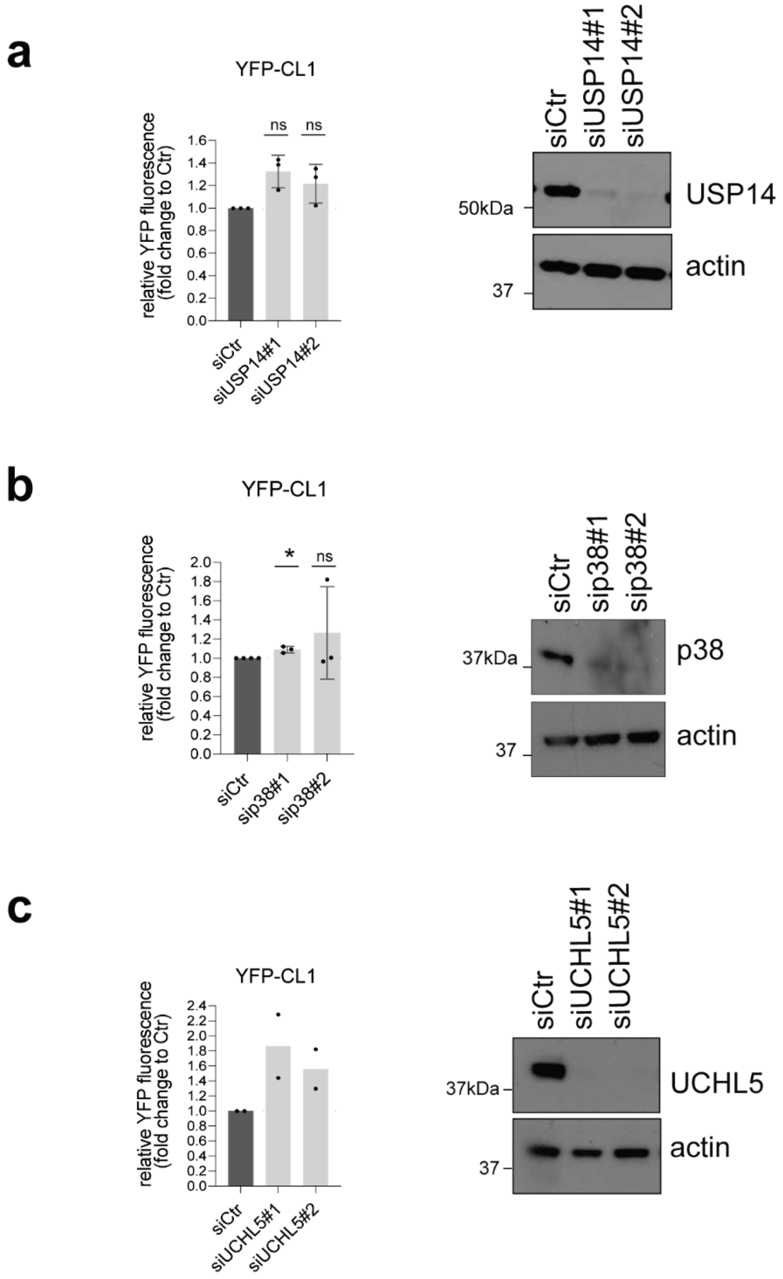

Supplementary Figure 3: (a-b) MelJuSo YFP-CL1 reporter cells were transiently transfected with siRNAs for 48 hours and analyzed by flow cytometry for YFP expression. (n=3 (a,b), mean ± SD, one-sample t-test, *P<0.05, ns: non-significant; n=2 (c)). Samples were also analyzed by western blotting for knockdown efficiencies of the siRNAs with the indicated antibodies.

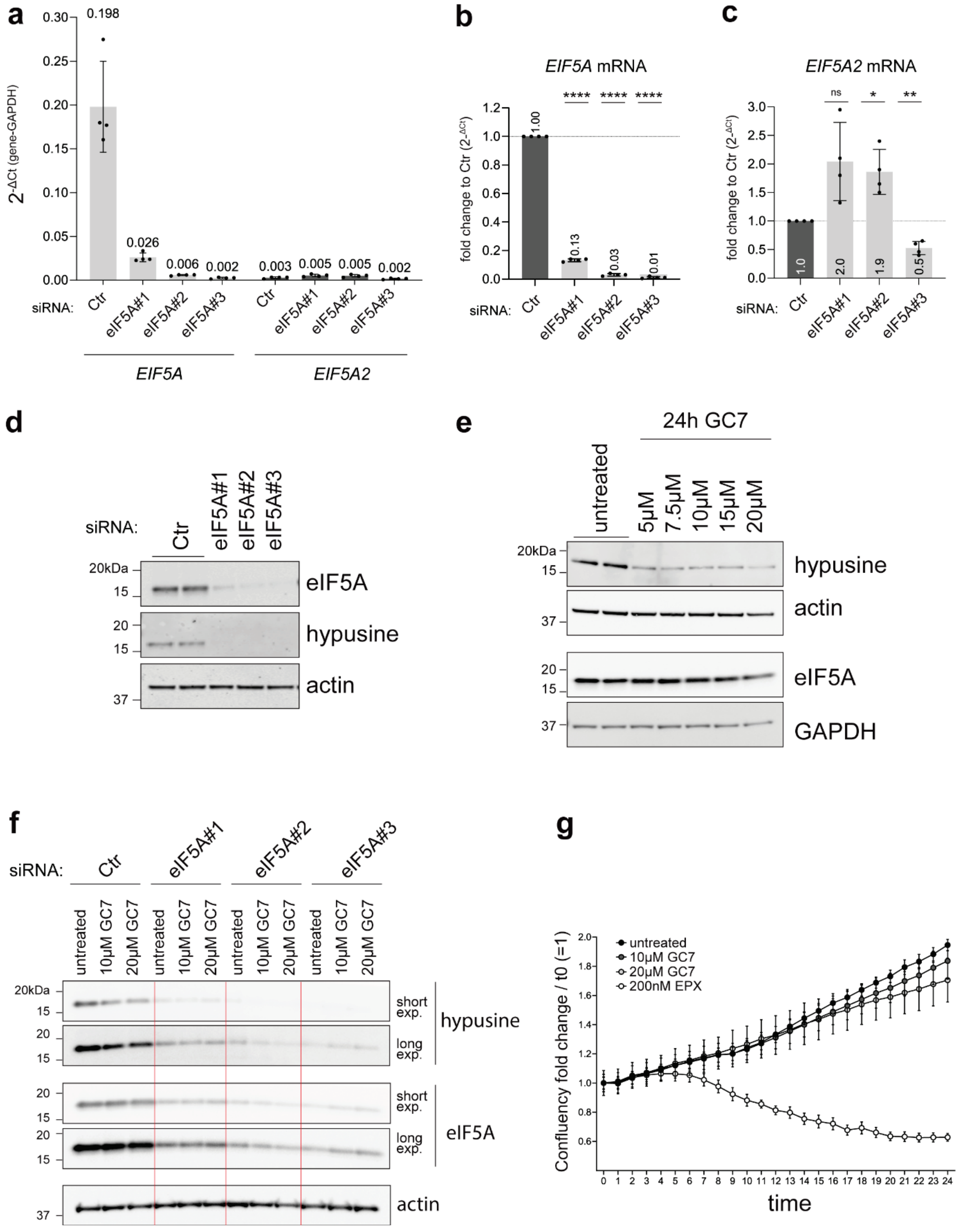

Supplementary Figure 4: (a-c) MelJuSo YFP-CL1 reporter cells were transiently transfected with an siRNAs targeting *EIF5A* and *EIF5A2* for 72 hours and analyzed by qRT-PCR for knockdown efficiency. Data are presented as (a) relative expression 2$^{\wedge(-\Delta Ct)}$ or as (b,c) fold change to their own control (n=4, mean ± SD, one-sample t-test, *P<0.05, **P<0.01, ****P<0.01, ns: non-significant). **(d)** MelJuSo YFP-CL1 reporter

cells were transiently transfected with siRNAs for 72 hours and analyzed by western blotting for knockdown efficiencies with the indicated antibodies. Relates to flow cytometry data from Fig. 4a. **(e)** MelJuSo YFP-CL1 reporter cells were treated for 24 hours with increasing concentrations of GC7 and analyzed by western blotting for hypusine depletion using indicated antibodies. Relates to flow cytometry data from Fig. 4c. **(f)** MelJuSo YFP-CL1 reporter cells were transiently transfected with siRNAs for 72 hours and treated the last 24 hours with two different concentrations of GC7. Samples were analyzed by western blotting for knockdown efficiencies and hypusine depletion with the indicated antibodies. Relates to flow cytometry data from Fig. 4f. **(g)** MelJuSo YFP-CL1 reporter cells were treated for 24 hours with two concentrations of GC7 or 200 nM of epoxomicin and monitored 24 hours for their growth curves and analyzed by confluency measurement (four images per condition, mean ± SD).

.

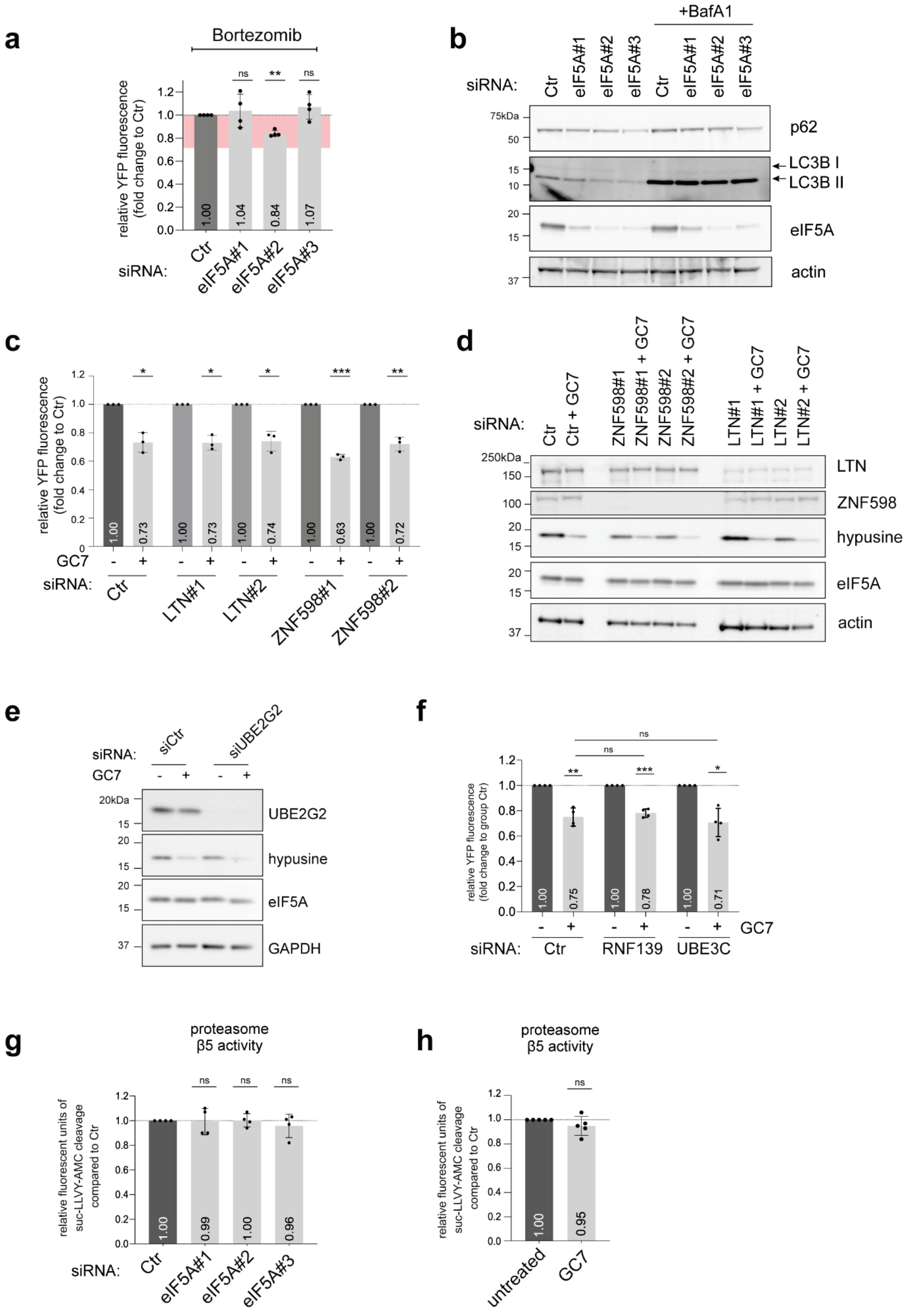
a
Bortezomib
relative YFP fluorescence (fold change to Ctr)
ns ** ns
1.00 1.04 0.84 1.07
siRNA: Ctr eIF5A#1 eIF5A#2 eIF5A#3
b
+BafA1
siRNA: Ctr eIF5A#1 eIF5A#2 eIF5A#3 Ctr eIF5A#1 eIF5A#2 eIF5A#3
75kDa 50 p62
15 10 LC3B I LC3B II
20 15 eIF5A
37 actin
c
relative YFP fluorescence (fold change to Ctr)
* * * *** **
1.00 0.73 1.00 0.73 1.00 0.74 1.00 0.63 1.00 0.72
GC7 - + - + - + - + - +
siRNA: Ctr LTN#1 LTN#2 ZNF598#1 ZNF598#2
d
siRNA: Ctr Ctr + GC7 ZNF598#1 ZNF598#1 + GC7 ZNF598#2 ZNF598#2 + GC7 LTN#1 LTN#1 + GC7 LTN#2 LTN#2 + GC7
250kDa 150 LTN
100 ZNF598
20 15 hypusine
20 15 eIF5A
37 actin
e
siRNA: siCtr siUBE2G2
GC7 - + - +
20kDa 15 UBE2G2
20 15 hypusine
20 15 eIF5A
37 GAPDH
f
relative YFP fluorescence (fold change to group Ctr)
ns ns ** *** *
1.00 0.75 1.00 0.78 1.00 0.71
- + - + - + GC7
siRNA: Ctr RNF139 UBE3C
g
proteasome β5 activity
relative fluorescent units of suc-LLVY-AMC cleavage compared to Ctr
ns ns ns
1.00 0.99 1.00 0.96
siRNA: Ctr eIF5A#1 eIF5A#2 eIF5A#3
h
proteasome β5 activity
relative fluorescent units of suc-LLVY-AMC cleavage compared to Ctr
ns
1.00 0.95
untreated GC7

Supplementary Figure 5: (a) MelJuSo YFP-CL1 cells were transfected with 20 nM siRNAs against eIF5A for 72 hours. The final 8 hours cells were incubated with 25 nM bortezomib and represented as a fold change to siCtr (n=4, mean ± SD, one-sample t-test, **P<0.01, ns: non-significant), relates to Fig. 5a. **(b)** MelJuSo YFP-CL1 cells were transfected with 20 nM siRNAs against eIF5A for 72 hours. The final 8 hours cells were incubated with 100 nM BafA1 and analyzed by western blotting using the indicated antibodies. **(c)** MelJuSo YFP-CL1 cells were transfected with 20 nM siRNAs against LTN1 or ZNF598 for 72 hours and treated the last 24 hours with 12 µM GC7 and represented as a fold change to its own control (n=3, mean ± SD, one-sample t-test, *P<0.05, **P<0.01, ***P<0.001). **(d)** Samples from (c) were analyzed by western blotting for knockdown efficiencies and hypusine depletion with the indicated antibodies. **(e)** MelJuSo YFP-CL1 reporter cells were transiently transfected with an siRNA directed against UBE2G2 for 72 hours, treated the last 24 hours with 12 µM GC7, and analyzed by western blotting for knockdown efficiencies and hypusine depletion with the indicated antibodies. Relates to flow cytometry data from Fig. 5B. **(f)** MelJuSo YFP-CL1 cells were transfected with 20 nM siRNAs against RNF139 or UbE3C for 72 hours and treated the last 24 hours with 12 µM GC7 and represented as a fold change to its own control (n=4, mean ± SD, one-sample t-test, *P<0.05, **P<0.01, ***P<0.001). **(g)** MelJuSo YFP-CL1 cells were transfected with 20 nM siRNAs against eIF5A for 72 hours and lysates were incubated with suc-Leu-Leu-Val-Tyr-AMC and monitored every minute for 60 min. Data are represented as the fold change at t= 30 min relative to its control (n=4, mean ± SD, one-sample t-test, ns: non-significant). **(h)** MelJuSo YFP-CL1 cells were treated with 10 µM GC7 for 24 hours and lysates were incubated with suc-Leu-Leu-Val-Tyr-AMC and monitored every minute for 60 min. Data are represented as the fold change at t= 30 min relative to its control (n=4, mean ± SD, one-sample t-test, ns: non-significant).

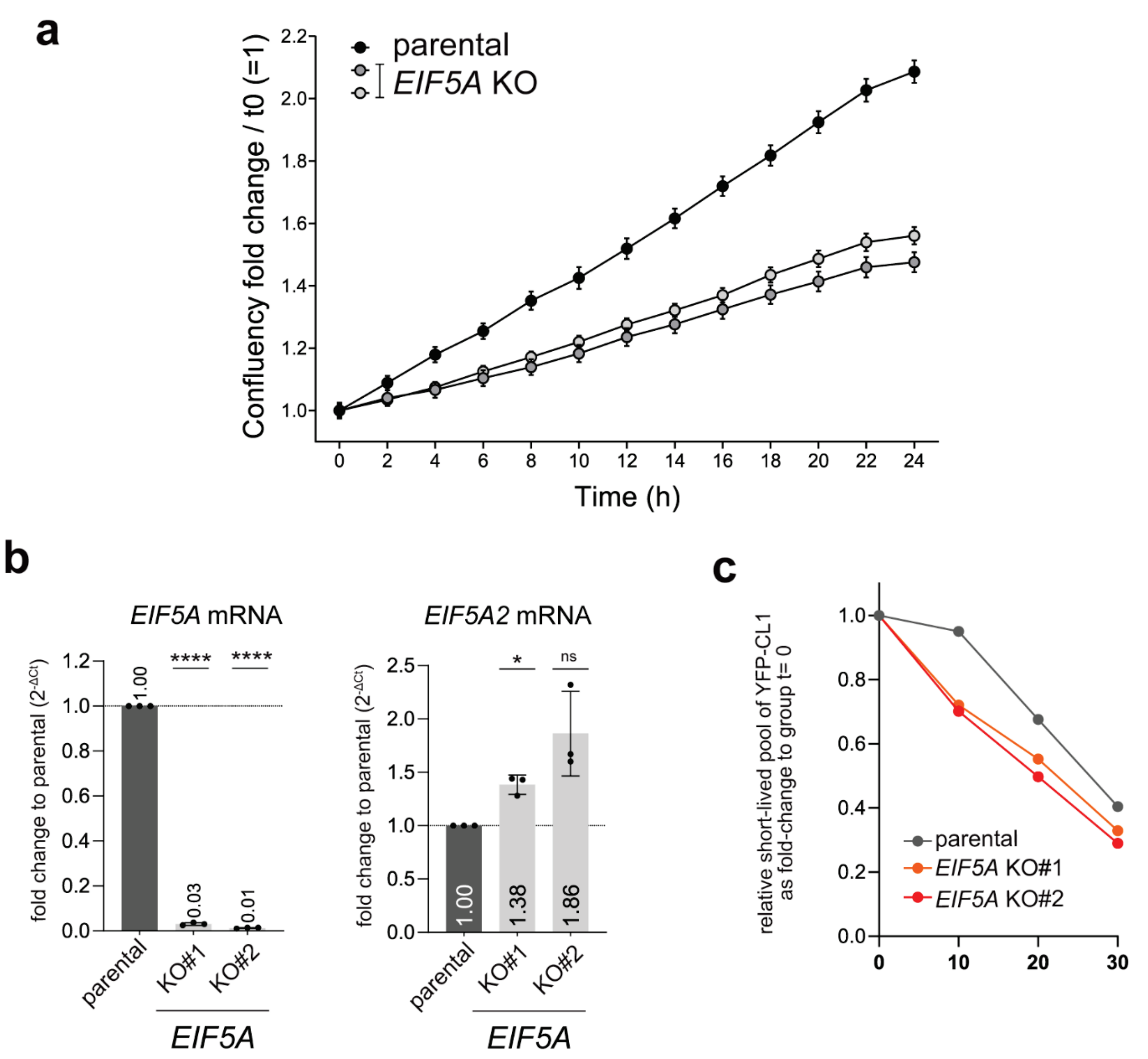

Supplementary Figure 6: (a) Two MelJuSo *EIF5A* KO cell lines and control cells were monitored 24 hours for their growth curves and analyzed by confluency measurement (four images per condition, mean ± SD). **(b)** Two MelJuSo *EIF5A* KO cell lines and control cells were analyzed by qRT-PCR for mRNA expression of *EIF5A* and *EIF5A2*. Data are presented as a fold change to its own control (n=3, mean ± SD, one-sample t-test, *P<0.05, ****P<0.01, ns: non-significant). **(c)** Two *EIF5A* KO cell lines and MelJuSo control cells were incubated with 20 µg/ml cycloheximide for the indicated times and subjected to flow cytometry analysis. The rate of short-lived YFP-CL1 was calculated as a subtraction from a four hours incubation time point and presented as the fold-change to its own control. One representative experiment out of three is shown.

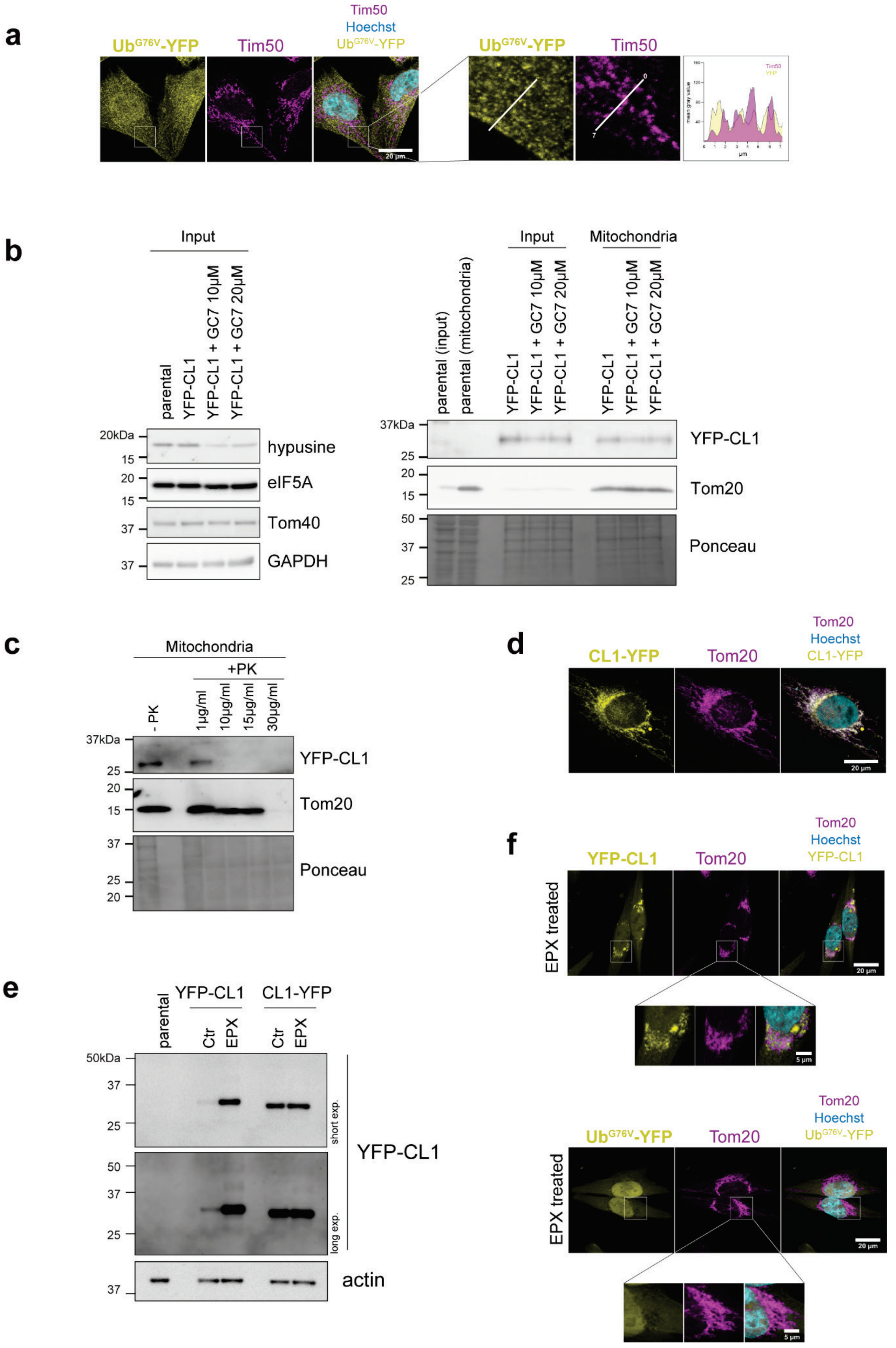
a
Ub G76V-YFP
Tim50
Tim50
Hoechst
Ub G76V-YFP
Ub G76V-YFP
Tim50
20 µm
b
Input
parental
YFP-CL1
YFP-CL1 + GC7 10µM
YFP-CL1 + GC7 20µM
20kDa
15
hypusine
20
15
eIF5A
37
Tom40
37
GAPDH
Input
Mitochondria
parental (input)
parental (mitochondria)
YFP-CL1
YFP-CL1 + GC7 10µM
YFP-CL1 + GC7 20µM
YFP-CL1
YFP-CL1 + GC7 10µM
YFP-CL1 + GC7 20µM
37kDa
25
YFP-CL1
20
15
Tom20
50
37
25
Ponceau
c
Mitochondria
+PK
- PK
1µg/ml
10µg/ml
15µg/ml
30µg/ml
37kDa
25
YFP-CL1
20
15
Tom20
37
25
20
Ponceau
d
CL1-YFP
Tom20
Tom20
Hoechst
CL1-YFP
20 µm
e
parental
YFP-CL1
CL1-YFP
Ctr
EPX
Ctr
EPX
50kDa
37
25
short exp.
50
37
25
long exp.
YFP-CL1
37
actin
f
EPX treated
YFP-CL1
Tom20
Tom20
Hoechst
YFP-CL1
20 µm
5 µm
EPX treated
Ub G76V-YFP
Tom20
Tom20
Hoechst
Ub G76V-YFP
20 µm
5 µm

Supplementary Figure 7: (a) MelJuSo Ub^{G76V}-YFP cells were stained for reporter levels using an anti-GFP antibody and for the mitochondrial network using an anti-Tim50 antibody and imaged by confocal microscopy. Co-localization was assessed by FiJi software analysis. Representative images, scale bar = 20 µm. Relates to Fig. 6a. **(b)** MelJuSo dual-fluorescent reporter cells were fractionated and 5 µg protein of each sample was analyzed by western blotting using anti-GFP for reporter expression, compartment specific antibodies anti-Tom40 or anti-Tom20. The input was analyzed additionally for inhibition of hypusination. **(c)** MelJuSo dual-fluorescent reporter cells were fractionated and treated without or with increasing concentrations of Proteinase K. 5 µg protein of each sample was analyzed by western blotting using anti-GFP and anti-Tom20 antibody. **(d)** CL1-YFP was overexpressed in MelJuSo cells for 48 hours, stained with an anti-Tom20 antibody and imaged by confocal microscopy. Representative images, scale bar = 20 µm. **(e)** YFP-CL1 and CL1-YFP were transiently overexpressed in MelJuSo cells for 48 hours and treated for the last 16 hours with 100 nM epoxomicin (EPX). Samples were analyzed by western blotting using anti-GFP for reporter expression. **(f)** MelJuSo YFP-CL1 and Ub^{G76V}-YFP cells were plated, treated the last 16 hours with 100 nM epoxomicin (EPX), stained with an anti-Tom20 antibody and imaged by confocal microscopy. Representative images, scale bar = 20 µm, zoom-in images: scale bar = 5 µm.

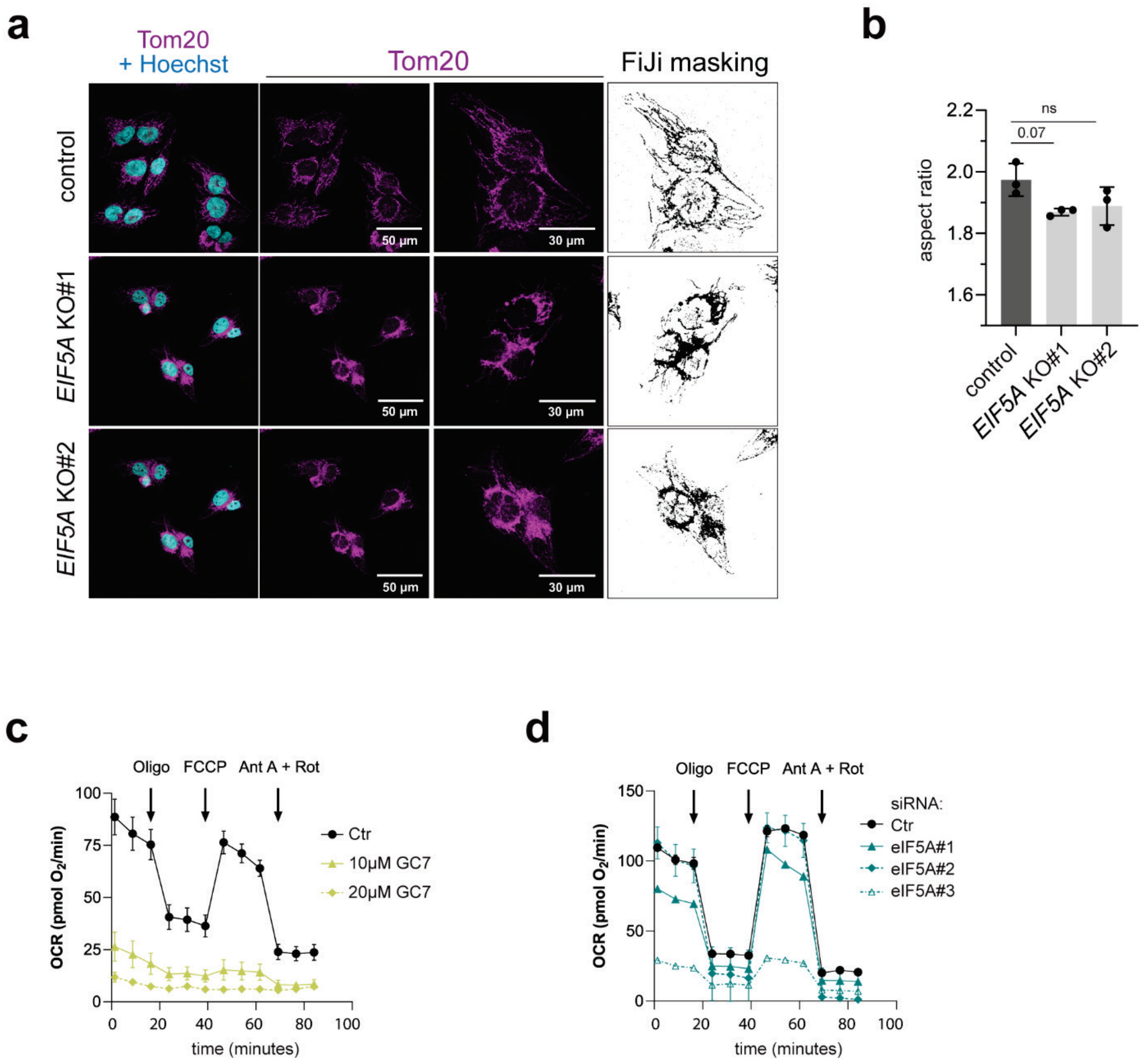

Supplementary Figure 8: (a) MelJuSo *EIF5A* KO and parental cells were stained for the mitochondrial network with an anti-Tom20 antibody and imaged by confocal microscopy. Representative images: scale bar = 50 µm, zoom-in images: scale bar = 30 µm. Deconvoluted images, as indicated by a representative cell, were used for staining quantifications. **(b)** Quantification of the aspect ratio (n= 3, mean ± SD, Kruskal-Wallis test, ns: non-significant). >37 cells were analyzed per cell line and experiment. **(c)** MelJuSo YFP-CL1 cells were treated with 10 µM and 20 µM GC7 for 24 hours and analyzed for their oxygen consumption rate using a Seahorse Analyzer. **(d)** MelJuSo YFP-CL1 cells were transfected with 20 nM siRNAs against eIF5A for 72 hours and analyzed for their oxygen consumption rate using a Seahorse Analyzer.

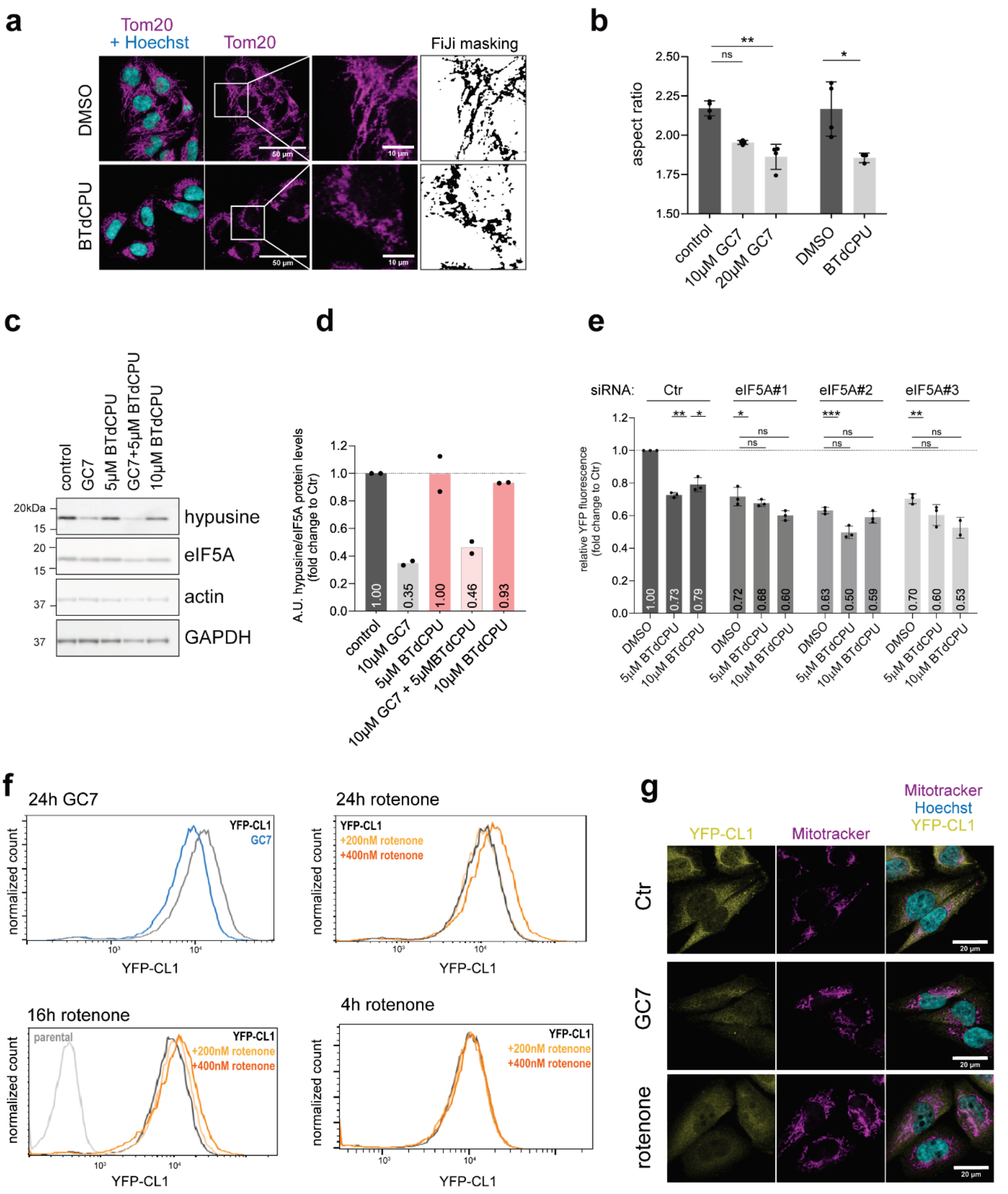

Supplementary Figure 9: (a) MelJuSo cells were treated with 10 µM BTdCPU for 24 hours, stained for the mitochondrial network with an anti-Tom20 antibody and imaged by confocal microscopy. Representative images, larger image: scale bar = 50 µm, zoom-in image: scale bar = 10 µm. Deconvoluted images, as indicated by a representative cell, were used for staining quantifications **(b)** Quantification of the aspect ratio (n= 4 for GC7 and n=3 for BTdCPU, mean ± SD, Kruskal-Wallis test, ns:

non-significant). Relates to Fig. 6d and S9a, >20 cells were analyzed per treatment and experiment. **(c)** MelJuSo YFP-CL1 cells were treated with 10 µM GC7, 5 µM and 10 µM BTdCPU or a combination of both compounds. Samples were analyzed by western blotting for hypusine depletion with the indicated antibodies. **(d)** Quantification of hypusine levels from western blots (n=2). **(e)** MelJuSo YFP-CL1 cells were transfected with 20 nM siRNAs against eIF5A for 72 hours, treated the last 24 hours with the 5 µM or 10 µM BTdCPU and analyzed by flow cytometry for YFP fluorescence. (n=3, mean ± SD, one sample t-test when compared to siCtr or Kruskal-Wallis test when samples were compared within each siEIF5A condition, *P<0.05, **P<0.001, ***P<0.0001, ns: non-significant). **(f)** MelJuSo YFP-CL1 cells were treated with 200 nM or 400 nM rotenone for the indicated time points and with 10 µM GC7 for 24h as a control. Samples were analyzed by flow cytometry and one representative histogram is shown of at least two independent experiments. Relates to Fig. 6g. **(g)** MelJuSo YFP-CL1 reporter cells were treated with 10 µM GC7 and 200 nM rotenone stained with an anti-GFP antibody and Mitotracker and imaged by confocal microscopy. Representative images, scale bar = 20 µm.

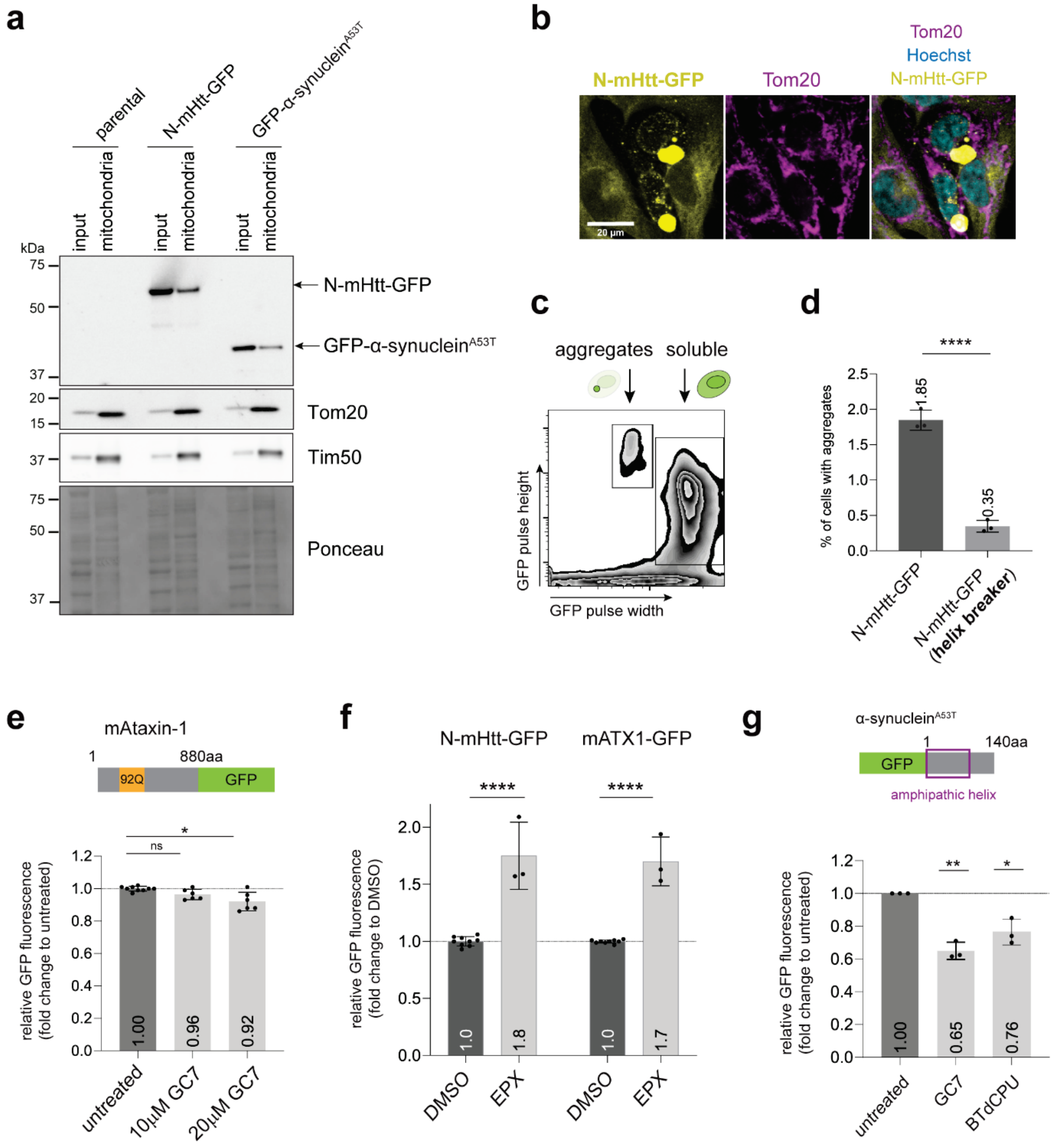

Supplementary Figure 10: (a) MelJuSo cells with stable expression of N-mHtt-GFP and GFP-α-synuclein^{A53T} were induced for 96h, fractionated for mitochondria and 5 µg protein of each sample was analyzed by western blotting using anti-GFP for N-mHtt-GFP and GFP-α-synuclein^{A53T} expression and compartment specific antibodies anti-Tim50 and anti-Tom20. **(b)** MelJuSo cells with inducible expression of N-mHtt-GFP were plated for 48 hours, stained with anti-Tom20 and imaged by confocal microscopy. Representative images, larger image: scale bar = 20 µm. **(c)** Schematics of PulSa assay to quantify N-mHtt-GFP aggregates in cells. **(d)** N-mHtt-GFP and a helix breaker mutant were transiently overexpressed in MelJuSo cells for 48 hours and the

percentage of cells with aggregates were analyzed by PulSa (n=3, mean ± SD, unpaired t-test, **P<0.01). **(e)** MelJuSo cells were transiently transfected with mATX1-GFP for 48 hours, treated the last 24 hours with 10 µM or 20 µM GC7 and analyzed by flow cytometry for GFP expression. (n=3 with three samples for control conditions and two samples for GC7 treated condition per replicate, mean ± SD, Kruskal-Wallis test, *P<0.05, ns: non-significant). **(f)** MelJuSo cells were transiently transfected with N-mutHtt-GFP and mATX1-GFP for 48 hours, treated the last 16 hours with 100 nM epoxomicin (EPX) and analyzed by flow cytometry for GFP expression (n=3 with three samples for control conditions and one sample for GC7 treated condition per replicate,, mean ± SD, unpaired t-test per transfected group, ****P<0.0001). Controls are the same as in Figure 8a and Supplementary. Figure 10e. **(g)** MelJuSo cells with inducible expression of GFP-α-synuclein^{A53T} were plated for 48 hours, treated the last 24 hours with 10 µM GC7 or 5 µM BTdCPU and analyzed by flow cytometry for GFP expression, (n=3, mean ± SD, one-sample t-test, *P<0.05, **P<0.01).

Supplementary Figure 11: Uncropped blots to Supplementary Figures S1b, S2a, S2c, S2d, S2f, S2g, S2i, S2j, S3a-c, S4d-f, S5b, S5d, S5e, S6b, S6c, S6e, S9c, S10a

Suppl. Fig. 1b

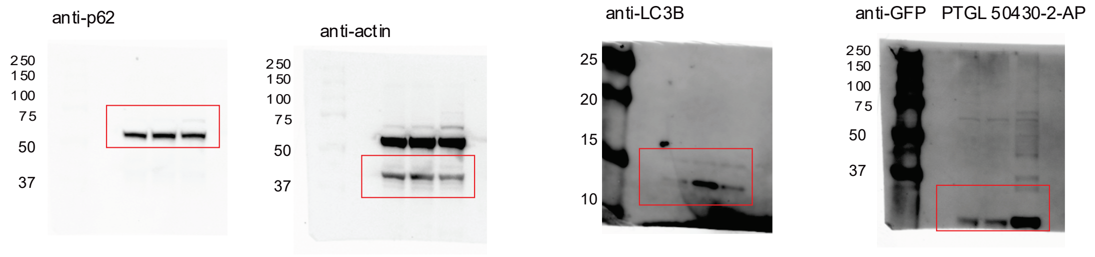

Suppl. Fig.2a

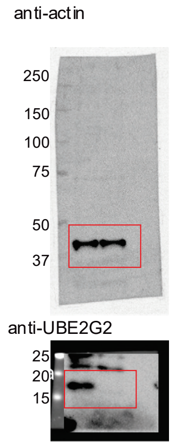

Suppl. Fig.2c

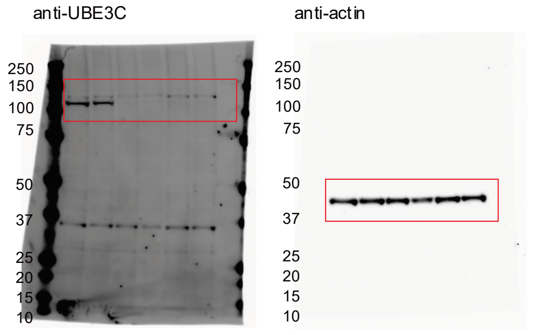

Suppl. Fig.2d

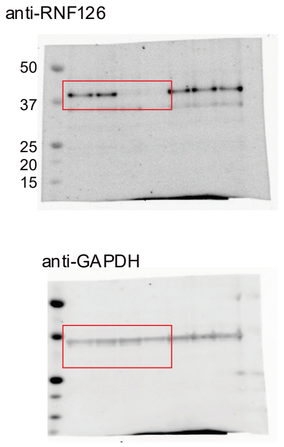

Suppl. Fig.2f

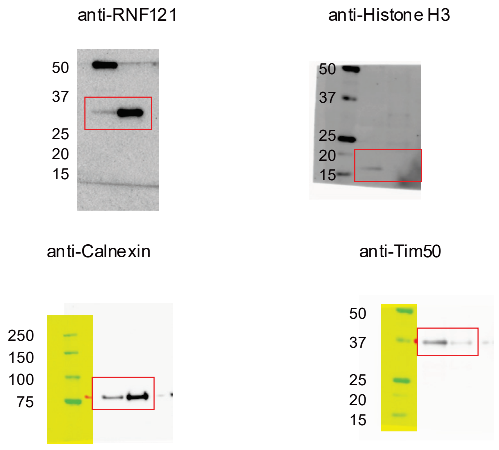

Suppl. Fig.2g

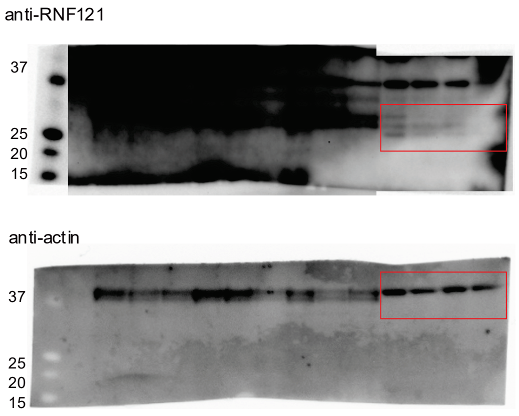

Suppl. Fig.2i

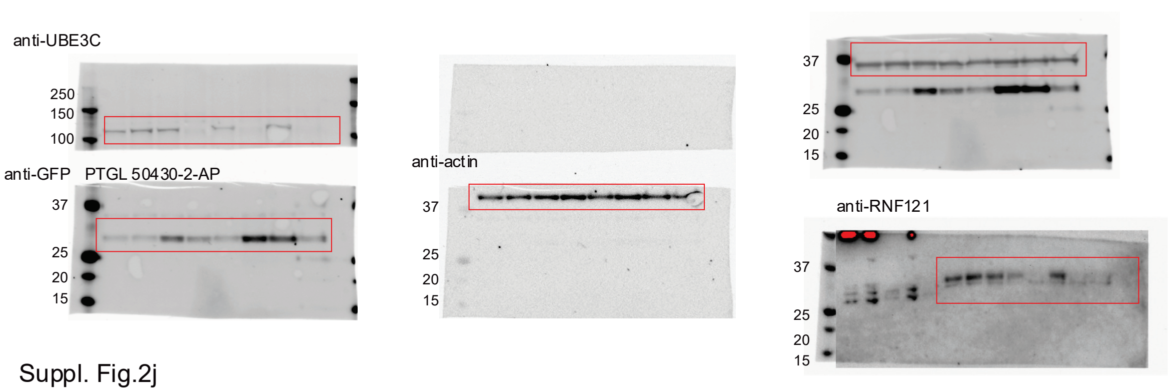

Suppl. Fig.2j

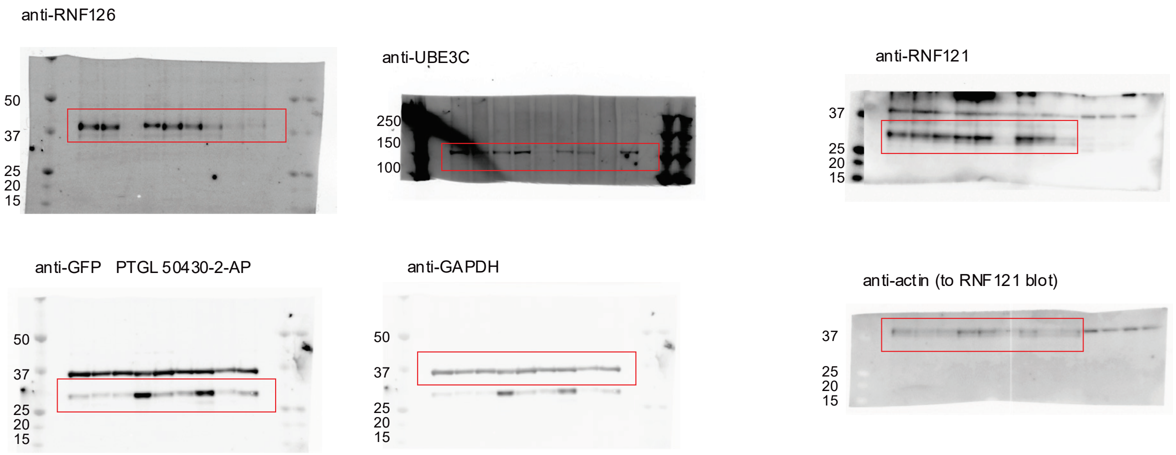

Suppl. Fig.3a-c

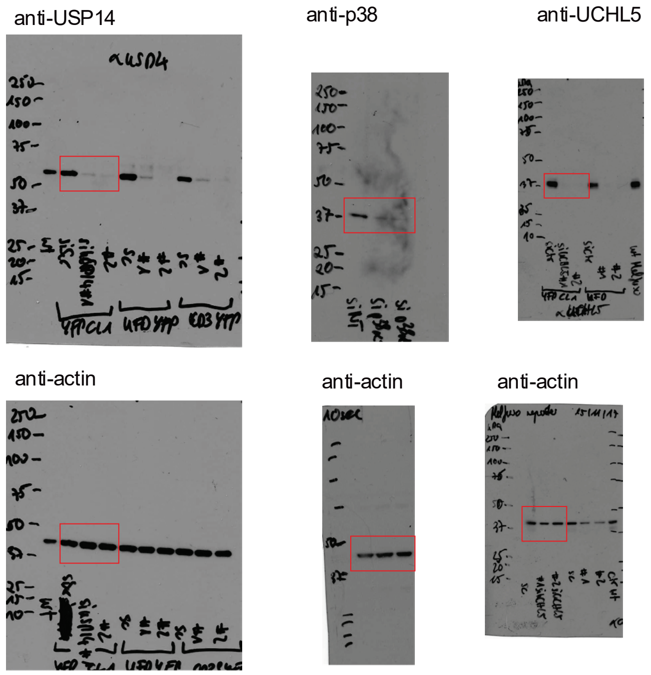

Suppl. Fig.4d

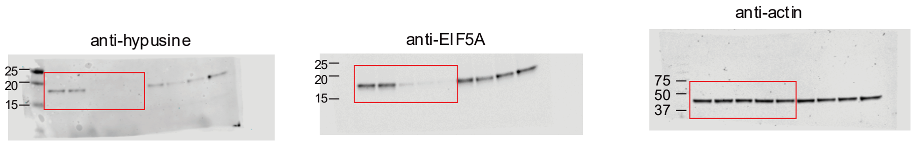

Suppl. Fig.4e

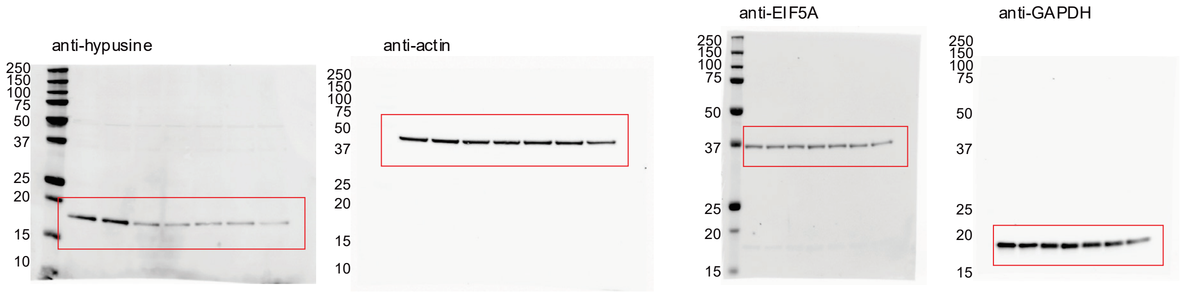

Suppl. Fig.4f

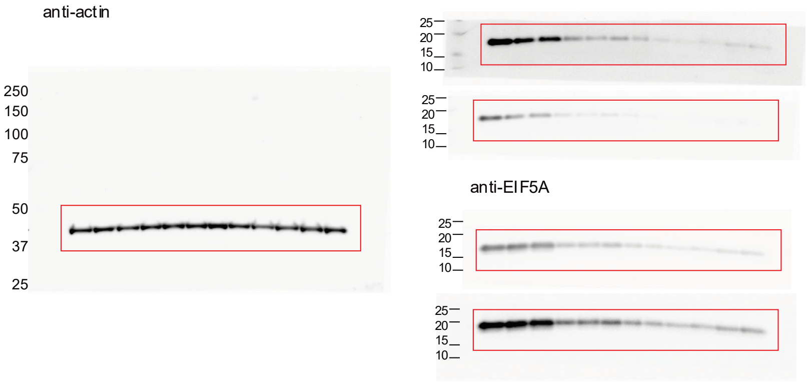

Suppl. Fig.5b

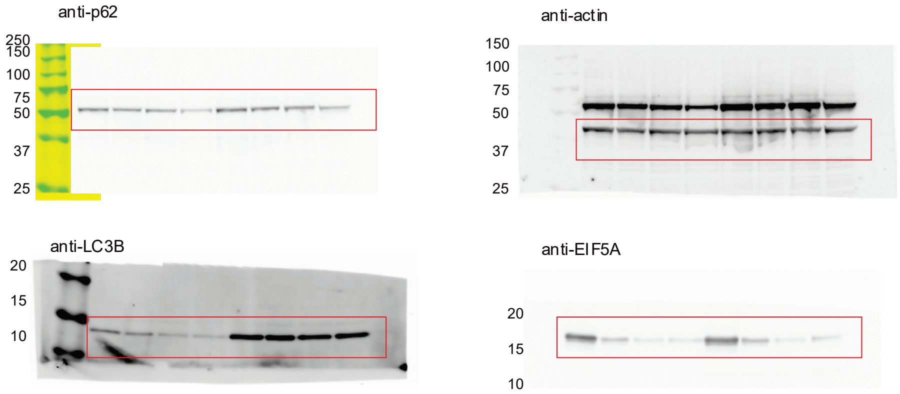

Suppl. Fig.5d

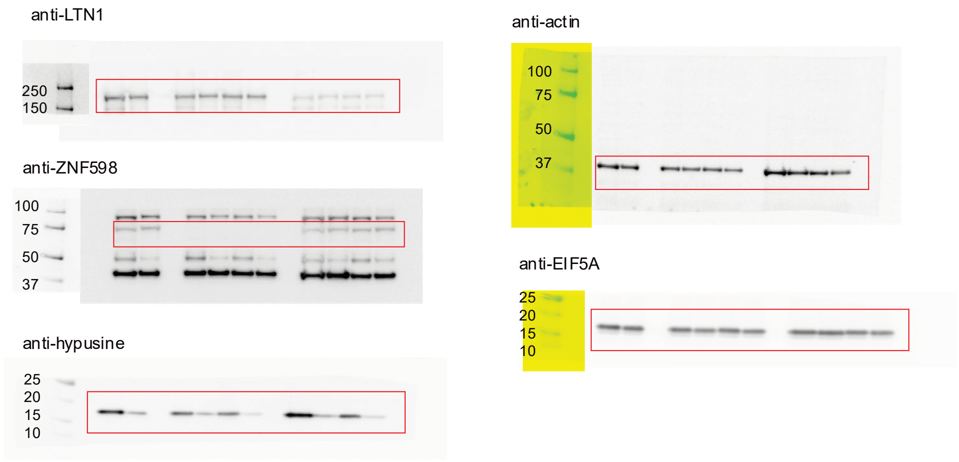

Suppl. Fig.5e

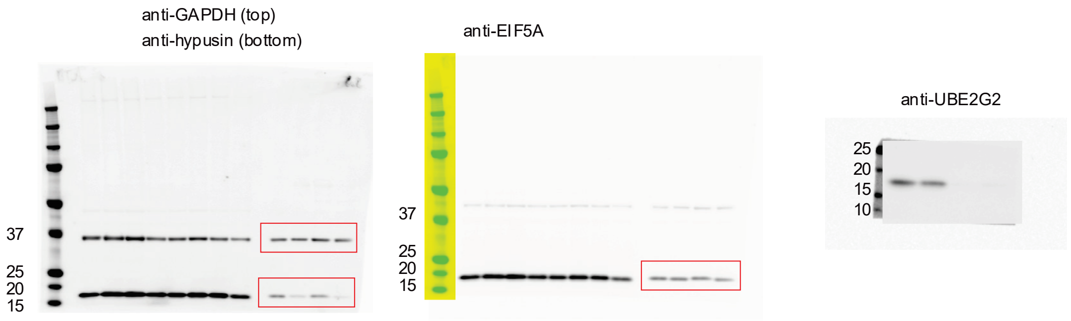

Suppl. Fig.7b

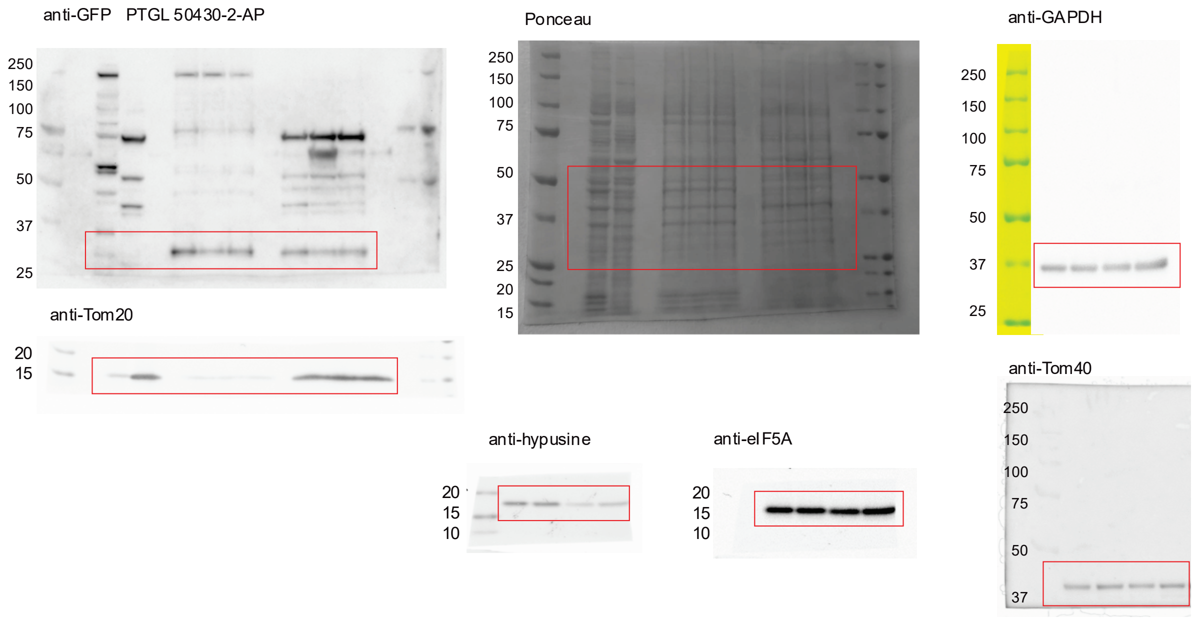

Suppl. Fig.7c

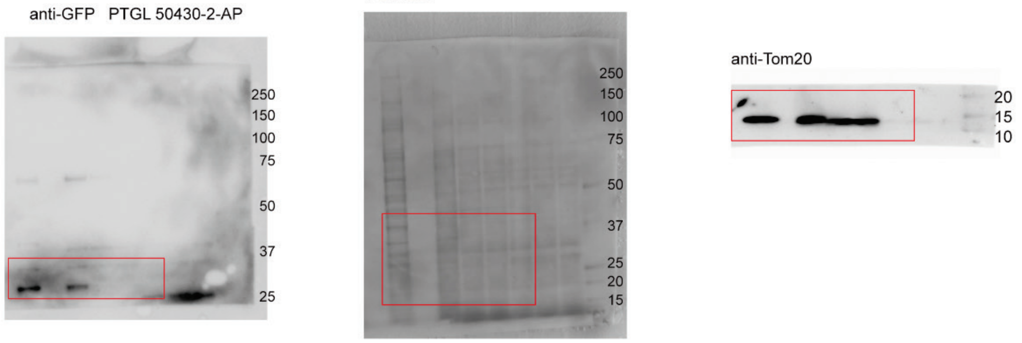

Suppl. Fig.7e

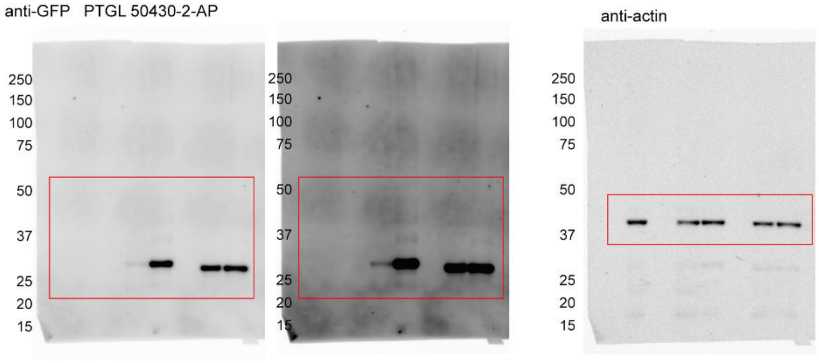

Suppl. Fig.9c

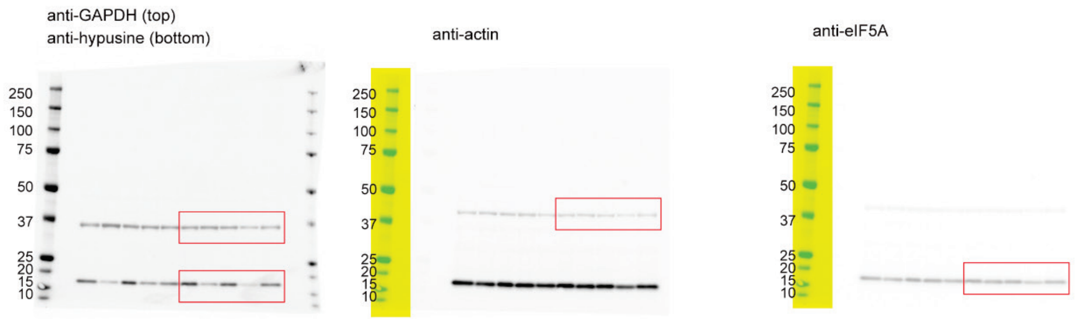

Suppl. Fig.10a

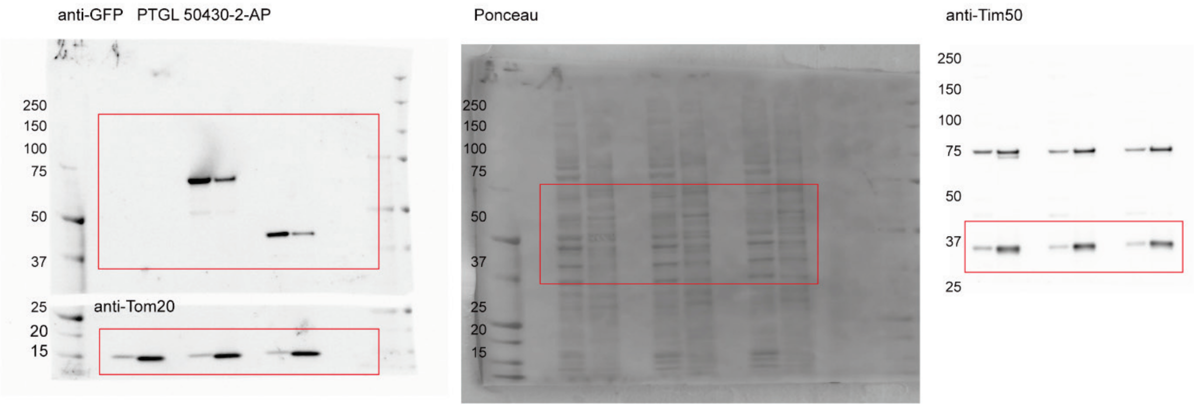

Supplementary Figure 12: Gating Strategy for flow cytometry data:

1. Stable expression of YFP-tagged proteins/reporter
   a. Living cells (SSC-A vs FSC-A)
   b. Singlets (FSC-A vs FSC-W)
   c. Median fluorescent intensity of all cells

a. b. c.

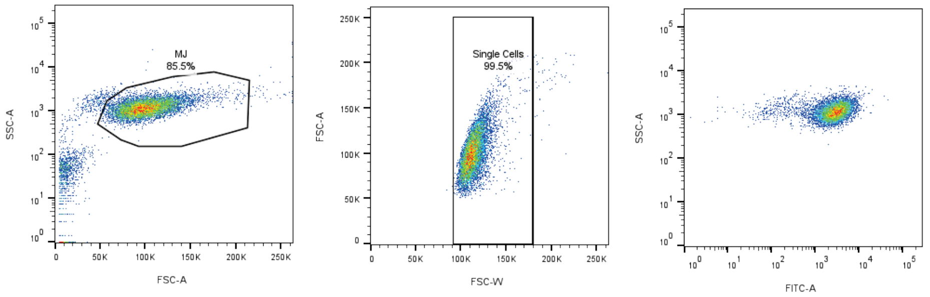

2. Stable expression of dual-fluorescent tagged reporter
   a. Living cells (SSC-A vs FSC-A)
   b. Singlets (FSC-A vs FSC-W)
   c. Median fluorescent intensity of all cells for all fluorescent colors

a. b. c.

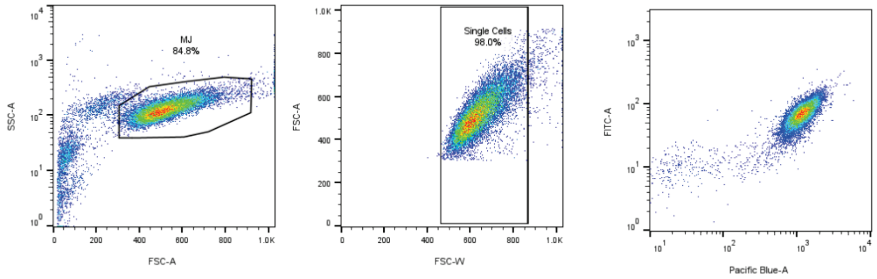

3. Transient expression of YFP/GFP-tagged proteins/reporter
   a. Living cells (SSC-A vs FSC-A)
   b. Singlets (FSC-A vs FSC-W)
   c. Positive cells (SSC-A vs. FITC-A) based on negative cells
   d. Median fluorescent intensity of all cells

a. b. c./d.

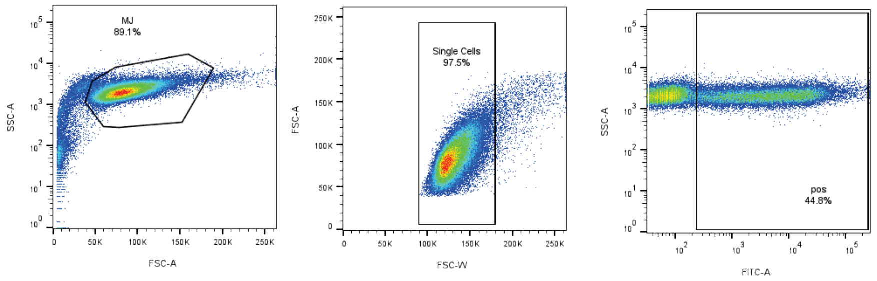

4. PulSa analysis of soluble expression/ aggregates
    a. Living cells (SSC-A vs FSC-A)
    b. Singlets (FSC-A vs FSC-W)
    c. If transient expression: Positive cells (SSC-A vs. FITC-A) based on negative cells
    d. PulSa (FITC-H vs. FITC-W)
    e. Median fluorescent intensity of soluble and aggregated fractions separately

a. b. c.-e.

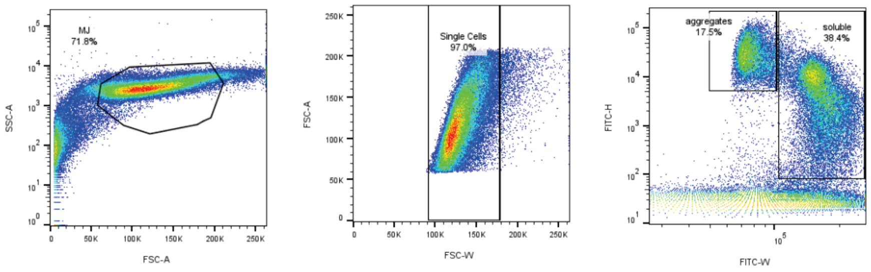

Supplementary Table S1

Criteria: ≥2 sgRNAs in both data sets and pos LFC in MAGeCK analysis

	replicate 1	replicate 2	total #
**YFP-CL1 high**	**shared R1-R2 (824)**	**shared R1-R2 (824)**	
average LFC	0.93	0.83	
	**additional R1 (257)**	**additional R2 (493)**	
average LFC	0.89	0.8	**1574**
**YFP-CL1 low**	**shared R1-R2 (1187)**	**shared R1-R2 (1187)**	
average LFC	0.67	0.62	
	**additional R1 (310)**	**additional R2 (580)**	
average LFC	0.65	0.62	**2077**

Supplementary Table 2

Antibodies	Supplier	Cat#	Used dilution
Actin	abcam	ab8226	WB 1:5000
Calnexin	Proteintech	66903-1-Ig	WB 1:1000
eIF5A	BD Biosciences	Clone 26/eIF-5a	WB 1:5000
GAPDH	abcam	ab9485	WB 1:5000
GFP	abcam	ab290	WB 1:5000
GFP	Proteintech	50430-2-AP	WB 1:5000
Hypusine	Merck/Millipore	ABS1064-I	WB 1:1000
LC3B	Merck/Millipore	L7543	WB 1:2000
LTN1	Proteintech	28452-1-AP	WB 1:1000
p38	CST	#9218	WB 1:1000
p62	BD Biosciences	BD 610832	WB 1:2000
PDH-E1α	Santa Cruz	BD Biosciences	WB 1:1000
RNF121	HumanProteinAtlas	HPA046041	WB 1:1000
RNF126	Proteintech	66647-1-Ig	WB 1:1000
Tim50	Santa Cruz	C-9, sc-393678	WB 1:1000, IF 1:500
Tom20	Santa Cruz	FL-145, sc11415	WB 1:1000, IF 1:500
tRFP	Evrogen	#AB233	WB 1:1000
UBE2G2	Proteintech	10722-1-AP	WB 1:5000
UBE3C	Bethyl Laboratories	A304-123A	WB 1:1000
ZNF598	abcam	AB241092	WB 1:1000
AlexaFluor 647	Invitrogen	Cat # A-21235	WB 1:2000
mouse HRP	CST	#7076	WB 1:10 000
mouse NIR	Li-Cor	IRDye® 800CW	WB 1:10 000
rabbit HRP	CST	#7074	WB 1:10 000
rabbit NIR	Li-Cor	IRDye® 680CW	WB 1:10 000

		Cat. Numbers /Assay IDs		
**siRNA**	**Supplier**	**Assay ID siRNA#1**	**Assay ID siRNA#2**	**Assay ID siRNA#3**
UBE2G2	Invitrogen, Silencer Select	s14584		
RNF139	Invitrogen, Silencer Select	s22178	s22179	
RNF121	Invitrogen, Silencer Select	s30675	s30677	
RNF126	Invitrogen, Silencer Select	s31185	s31186	
UBE3C	Invitrogen, Silencer Select	s18659	s18661	
EIF5A	Invitrogen, Silencer Select	s4594	s4596	s4595
Negative control No.2	Invitrogen, Silencer Select	cat#4390846		

siRNA sub-library:		Assay ID siRNA#1	Assay ID siRNA#2	Assay ID siRNA#3
BOLA3	Invitrogen, Silencer Select	s52323	s52324	s52322
HPF1;C4orf27	Invitrogen, Silencer Select	s29881	s29883	s29882
CMTM2	Invitrogen, Silencer Select	s44834	s44833	s44835
COMMD10	Invitrogen, Silencer Select	s28085	s28086	s28084
EIF5A	Invitrogen, Silencer Select	s4594	s4596	s4595
ENY2	Invitrogen, Silencer Select	s32447	s32449	s32448
FAM181B	Invitrogen, Silencer Select	s47896	s47895	s47897
GORAB	Invitrogen, Silencer Select	s40928	s40927	s535077
MAP3K7	Invitrogen, Silencer Select	s13766	s13768	s13767
NIF3L1	Invitrogen, Silencer Select	s229839	s34098	s229840
PLAT	Invitrogen, Silencer Select	s10608	s10609	s10607
RLIM	Invitrogen, Silencer Select	s27519	s27520	s27518
SLC11A2	Invitrogen, Silencer Select	s9710	s9708	s9709
CCDC187;MGC50722	Invitrogen, Silencer Select	s53157	s53156	s53158

gRNA, Assay ID	Supplier
EIF5A, CRISPR288707_SGM	Invitrogen, TrueGuide synthetic gRNA

Assays on demand	Supplier
Eif5a Hs00744729_s1	Applied Biosystems
Eif5a2 Hs00702673_s1	Applied Biosystems
Rnf139 Hs00183680_m1	Applied Biosystems
Rnf121 Hs01553223_m1	Applied Biosystems
Gapdh Hs02786624_g1	Applied Biosystems
Actb Hs01060665_g1	Applied Biosystems

Plasmids	Source
pCMV-YFP-CL1	Menéndez-Benito et al. 2005, PMID: 16103128
pCMV-YFP-CL1*	this study
pCMV-CL1-YFP	this study
pEF1α-tBFP-H2A-p2A-YFP-CL1	this study
pLentiCRISPR-tagBFP	addgene #75160
Cas9	CRISPR Functional Genomics Infrastructure Unit, Karolinska Institutet
N-mHtt-GFP	addgene#111730
N-mHtt-GFP helix breaker (HB)	this study
tet-off N-mHtt-GFP	this study
GFP-α-synuclein A53T	addgene #40823
tet-off GFP-α-synuclein A53T	this study
mAtaxin-1 GFP	this study

Primer site directed mutagenesis	
YFP-CL1* FW primer	CTCTTCTTTGTCTCACGCCGCTGCCCACGCGTGAGGATCCACCGGATC
YFP-CL1* RS primer	GATCCGGTGGATCCTCACGCGTGGGCAGCGGCGTGAGACAAAGAAGAG
mHtt-GFP helixbreaker FW primer	TGGCCACCCTGGAAAAACCGATGCCAGCATTTGAAAGCCTGAAAAG
mHtt-GFP helixbreaker RS primer	CTTTTCAGGCTTTCAAATGCTGGCATCGGTTTTTCCAGGGTGGCCATTTGG

**Primer for NEBuilder cloning**	
cerulean-H2A fragment into YFP-CL1 plasmid FW	CAAACGGGATCCACCGGATCTAGATAACGGGAGCGG
cerulean-H2A fragment into YFP-CL1 plasmid RS	TATCTAGATCCGGTGGATCCCGTTTGCC
GSG-p2A oligo FW	GATCTAGATAAC GGGAGCGGAGCTACTAACTTCAGCCTGCTGAAGCAGGCTGGCGACGTGGAGGAGAACCCTGGACCT
cerulean exchanged with tBFP FW	CTAGAGCGGCCGCATGGGCAGCGAGCTGATTAAG
cerulean exchanged with tBFP RS	CGAGATCTGAGTAATTAAGCTTGTGCCCCAGTTTG
pCMV echange with pEF1a FW	CGTATTACCGCCATGCATTAGTTATTAATGAGTAATTCATACAAAAGGACTCG
pCMV echange with pEF1a RS	ATTCGAAGCTTGAGCTCGAGATCTGAATTAAGCTTGTGCCCCAGTTTG
blasticidin exchange with puromycin in pTRE FW	ATGTATCTTATTGCGCCTTTTCCAAGGCAGC
blasticidin exchange with puromycin in pTRE RS	CCTCTACCTTCGGCACCGGGGGCACCGGGCTTGCGGGT
pTRE FW	CCCGGTGCCGAAGGTAGAG
pTRE RS	GGTGCTAGCCAATTCTCCAG
N-mHtt-GFP into pTRE FW	CAGATCGCCTGGAGAATTGGACCGCCACCATGGCGACCCTGGAAAAGC
N-mHtt-GFP into pTRE RS	ATCCCGGGCCCGCGGTACCGTTACTTGTACAGCTCGTCCATGCC
GFP-α-synuclein A53T into pTRE FW	CTGGAGAATTGGCTAGCACCGCCACCATGGTGAGCAAG
GFP-α-synuclein A53T into pTRE RS	AAAGGCGCAATAAGATACATTGATGAGTTTGGACAAAC

**Primer for CRISPR screen**	
Oligo pool	CTTGTGGAAAGGACGAAACACCGNNNNNNNNNNNNNNNNNNNNGTTTAAGAGCTAGAAATAGCAAGTTTAAATAAGGCT
Ultramer_RSL	TTTGTCTCAAGATCTAGTTACGCCAAGCTTNNNNNNNNNNGTGACTGGAGTTCAGACGTGTGCTCTTCCGATCAAAAAAGCACCGACTC<br>GGTGCCACTTTTTCAAGTTGATAACGGACTAGCCTTATTTAAACTTGCTATTTCTAGCTC
ds_fw	GGCTTTATATATCTTGTGGAAAGGACGAAACACCG
ds_rev	TTTGTCTCAAGATCTAGTTACGCCAAGC
Final insert	GGCTTTATATATCTTGTGGAAAGGACGAAACACCGNNNNNNNNNNNNNNNNNNNNGTTTAAGAGCTAGAAATAGCAAGTTTAAATAAGG<br>CTAGTCCGTTATCAACTTGAAAAAGTGGCACCGAGTCGGTGCTTTTTTGATCGGAAGAGCACACGTCTGAACTCCAGTCACNNNNNNN<br>NNNAAGCTTGGCGTAACTAGATCTTGAGACAAA
PCR1_fw	GGACTATCATATGCTTACCGTAACTTGAAAGTATTTCG
PCR1_rev	CTTTAGTTTGTATGTCTGTTGCTATTATGTCTACTATTCTTTCC
PCR2_fw	ACACTCTTTCCCTACACGACGCTCTTCCGATCTCTTGTGGAAAGGACGAAACAC
PCR2_rev	AGAAGACGGCATACGAGATCTGCCATTTGTCTCAAGATCTAGTTAC
PCR3_fw	AATGATACGGCGACCACCGAGATCTACAC[i5]ACACTCTTTCCCTACACGACGCTCT
PCR3_rev	CAAGCAGAAGACGGCATACGAGATCTGCCATTTG
CRISPRSeq	CGATCTCTTGTGGAAAGGACGAAACACCG

**Chemicals**	**Supplier/ Cat. Numbers**	**Treatment concentrations**
ATP	Sigma-Aldrich #1191	1mM
BafA1	MedChemExpress, HY-100558	100nM
Bortezomib	MedChemExpress, HY-10227	25nM
BTdCPU	MedChemExpress, HY-118266	5-10µM
CB-5083	Biovision, #BIOVB1032-5	10µM
Cycloheximide	Sigma-Aldrich, C4859	20mg/ml
DMSO	Sigma-Aldrich, D8418	-
Doxycycline	Sigma-Aldrich, D1822	0.1ng/µl
Epoxomicin	Sigma-Aldrich, #324801	100-500nM
GC7	Sigma-Aldrich, #259545	5-20µM
Hoechst 33342	Invitrogen/ Molecular Probes	1:5000
Rotenone	Sigma-Aldrich, #557368	200-400nM
TAK-243	MedChemExpress, HY-100487	1µM

**Buffer**	**Recipe**
2xLDS buffer complete	4x LDS sample buffer, 1x protease inhibitor, 1x NuPage reducing agent, 10uM MG132, 5mM NEM, in PBS
RIPA buffer complete	NaCl 150mM, Tris 50mM, NP40 1%, SDS 0.1%, Na-deoxycholate 0.5%, MG132 10uM, NEM 5mM
BFB1 buffer	225 mM mannitol,75 mM sucrose, 0.5 mM EGTA, 0.5% bovine serum albumin, 30 mM Tris–HCl, pH 7.4 in bi-distilled water, +10uM MG132
BFB2 buffer	225 mM mannitol, 75 mM sucrose, 0.5% (w/v) bovine serum albumin, 30 mM Tris–HCl, pH 7.4 in bi-distilled water +10uM MG132
BFB3 buffer	225 mM mannitol, 75 mM sucrose, 30 mM Tris–HCl, pH 7.4. Prepare 1 day before the experiment using bi-distilled water
BFB4 buffer	250 mM mannitol, 0.5 mM EGTA, 5 mM HEPES/KOH, pH 7.4 in bi-distilled water
Percoll dilution buffer	225 mM mannitol, 1 mM EGTA, 25 mM HEPES/KOH, pH 7.4 in bi-distilled water
proteasome activity lysis buffer	25 mM HEPES pH 7.2, 50 mM NaCl, 1 mM MgCl2, 1 mM ATP, 1 mM DTT, 10% glycerol, 0.5% Triton X-100
proteasome activity reaction buffer	25 mM HEPES pH 7.2, 50 mM NaCl, 1 mM MgCl2, 1 mM ATP, 1 mM DTT, 10% glycerol

**Commercial assays**	**Supplier/ Cat. Numbers**
QIAprep Spin Miniprep	Qiagen, #27104
QiAquick gel extraction	Qiagen, #28706
RNeasy Plus Mini kit	Qiagen, #74134

**SDS-PAGE**	**Supplier/ Cat. Numbers**
NuPAGE™ 4-12% Bis-Tris Protein Gels, 1.5 mm, 15-wells	Thermo Scientific, NP0336BOX
NuPAGE™ 4-12% Bis-Tris Protein Gels, 1.0 mm, 15-wells	Thermo Scientific, NP0323BOX

**Software**	**Source/ company**
Fiji ImageJ	Schindelin et al. Nat Methods 2012
FlowJo v10	BD Biosciences, GraphPad Software
ImageLab Software	Bio-Rad
Image Studio Light	LI-COR
Snapgene	SnapGene software (www.snapgene.com)
Metascape	Zhou et al. Nature Commun. 2019 10(1):1523
Seahorse XF Cell Mito Stress Test, Wave software v2.6.1	Agilent
