# Supplementary material for: Mitochondria serve as a holdout compartment for aggregation-prone proteins hindering efficient degradation
Source: Nat Commun. 2026 May 7;17:4195. doi: 10.1038/s41467-026-72783-0 (PMC13153185; doi:10.1038/s41467-026-72783-0)
Supplement: Supplementary file 2 — Description of Additional Supplementary Files [file 41467_2026_72783_MOESM2_ESM.pdf]

Description of Additional Supplementary Files

File Name: Supplementary Data 1

Description: MAGeCK analysis
