# Supplementary material for: Mitochondria serve as a holdout compartment for aggregation-prone proteins hindering efficient degradation
Source: Nat Commun. 2026 May 7;17:4195. doi: 10.1038/s41467-026-72783-0 (PMC13153185; doi:10.1038/s41467-026-72783-0)
Supplement: Supplementary file 4 — Reporting Summary [file 41467_2026_72783_MOESM4_ESM.pdf]

nature portfolio

Corresponding author(s): Nico Dantuma

Last updated by author(s): 29/12/2025

Reporting Summary

Nature Portfolio wishes to improve the reproducibility of the work that we publish. This form provides structure for consistency and transparency in reporting. For further information on Nature Portfolio policies, see our Editorial Policies and the Editorial Policy Checklist.

Statistics

For all statistical analyses, confirm that the following items are present in the figure legend, table legend, main text, or Methods section.

n/a	Confirmed	
☐	☒	The exact sample size (*n*) for each experimental group/condition, given as a discrete number and unit of measurement
☒	☐	A statement on whether measurements were taken from distinct samples or whether the same sample was measured repeatedly
☐	☒	The statistical test(s) used AND whether they are one- or two-sided<br>*Only common tests should be described solely by name; describe more complex techniques in the Methods section.*
☒	☐	A description of all covariates tested
☐	☒	A description of any assumptions or corrections, such as tests of normality and adjustment for multiple comparisons
☐	☒	A full description of the statistical parameters including central tendency (e.g. means) or other basic estimates (e.g. regression coefficient) AND variation (e.g. standard deviation) or associated estimates of uncertainty (e.g. confidence intervals)
☒	☐	For null hypothesis testing, the test statistic (e.g. *F*, *t*, *r*) with confidence intervals, effect sizes, degrees of freedom and *P* value noted<br>*Give P values as exact values whenever suitable.*
☒	☐	For Bayesian analysis, information on the choice of priors and Markov chain Monte Carlo settings
☒	☐	For hierarchical and complex designs, identification of the appropriate level for tests and full reporting of outcomes
☒	☐	Estimates of effect sizes (e.g. Cohen's *d*, Pearson's *r*), indicating how they were calculated

Our web collection on statistics for biologists contains articles on many of the points above.

Software and code

Policy information about availability of computer code

Data collection	ImageLab Software, Image Studio Light, BD FACSDiva Software, Incucyte® Acquisition Software, LAS X for Stellaris Acquisition Software, NIS Elements Acquisition Software Nikon CrEST X-Light V3, Seahorse XF Analyzer
Data analysis	FiJi ImageJ, FlowJo, GraphPad Prism, ImageLab Software, Image Studio Light, SnapGene, Metascape, Incucyte® Acquisition & Analysis Software, Seahorse XF Cell Mito Stress Test, Wave software

For manuscripts utilizing custom algorithms or software that are central to the research but not yet described in published literature, software must be made available to editors and reviewers. We strongly encourage code deposition in a community repository (e.g. GitHub). See the Nature Portfolio guidelines for submitting code & software for further information.

Data

Policy information about availability of data

All manuscripts must include a data availability statement. This statement should provide the following information, where applicable:

- Accession codes, unique identifiers, or web links for publicly available datasets
- A description of any restrictions on data availability
- For clinical datasets or third party data, please ensure that the statement adheres to our policy

Included in manuscript

Research involving human participants, their data, or biological material

Policy information about studies with human participants or human data. See also policy information about sex, gender (identity/presentation), and sexual orientation and race, ethnicity and racism.

Reporting on sex and gender	Experiments were performed in MelJuSo, which is a human melanoma cells derived from a Caucasian female.
Reporting on race, ethnicity, or other socially relevant groupings	Experiments were performed in MelJuSo, which is a human melanoma cells derived from a Caucasian female.
Population characteristics	N.A.
Recruitment	N.A.
Ethics oversight	N.A.

Note that full information on the approval of the study protocol must also be provided in the manuscript.

Field-specific reporting

Please select the one below that is the best fit for your research. If you are not sure, read the appropriate sections before making your selection.

[x] Life sciences [ ] Behavioural & social sciences [ ] Ecological, evolutionary & environmental sciences

For a reference copy of the document with all sections, see nature.com/documents/nr-reporting-summary-flat.pdf

Life sciences study design

All studies must disclose on these points even when the disclosure is negative.

Sample size	Quantifications are from at least three independent experiments or the indicated number of cells.
Data exclusions	No data were excluded.
Replication	All experiments have been replicated at least three times.
Randomization	Samples were handled as much as possible randomly.
Blinding	The researchers performing the experiments were not blinded for the sample identities.

Reporting for specific materials, systems and methods

We require information from authors about some types of materials, experimental systems and methods used in many studies. Here, indicate whether each material, system or method listed is relevant to your study. If you are not sure if a list item applies to your research, read the appropriate section before selecting a response.

Materials & experimental systems

n/a	Involved in the study	
[ ]	[x]	Antibodies
[ ]	[x]	Eukaryotic cell lines
[x]	[ ]	Palaeontology and archaeology
[x]	[ ]	Animals and other organisms
[x]	[ ]	Clinical data
[x]	[ ]	Dual use research of concern
[x]	[ ]	Plants

Methods

n/a	Involved in the study	
[x]	[ ]	ChIP-seq
[ ]	[x]	Flow cytometry
[x]	[ ]	MRI-based neuroimaging

Antibodies

Antibodies used	actin, Calnexin, eIF5A, GAPDH, GFP, hypusine, LC3B, LTN1, p38, p62, PDH-E1α, RNF121, RNF126, Tim50, Tom20, tRFP, UBE2G2, UBE3C, ZNF598
Validation	All antibodies are commercially available. Suppliers are mentioned in manuscript. Antibodies have not been independently validated by authors.

Eukaryotic cell lines

Policy information about cell lines and Sex and Gender in Research

Cell line source(s)	MelJuso cells (human melanoma cells) (gift Netherlands Cancer Institute)
Authentication	Cells have been authenticated (Sept 2024)
Mycoplasma contamination	Cells are frequently PCR tested on mycoplasma.
Commonly misidentified lines (See ICLAC register)	N.A.

Plants

Seed stocks	*Report on the source of all seed stocks or other plant material used. If applicable, state the seed stock centre and catalogue number. If plant specimens were collected from the field, describe the collection location, date and sampling procedures.*
Novel plant genotypes	*Describe the methods by which all novel plant genotypes were produced. This includes those generated by transgenic approaches, gene editing, chemical/radiation-based mutagenesis and hybridization. For transgenic lines, describe the transformation method, the number of independent lines analyzed and the generation upon which experiments were performed. For gene-edited lines, describe the editor used, the endogenous sequence targeted for editing, the targeting guide RNA sequence (if applicable) and how the editor was applied.*
Authentication	*Describe any authentication procedures for each seed stock used or novel genotype generated. Describe any experiments used to assess the effect of a mutation and, where applicable, how potential secondary effects (e.g. second site T-DNA insertions, mosiacism, off-target gene editing) were examined.*

Flow Cytometry

Plots

Confirm that:

- [x] The axis labels state the marker and fluorochrome used (e.g. CD4-FITC).
- [x] The axis scales are clearly visible. Include numbers along axes only for bottom left plot of group (a 'group' is an analysis of identical markers).
- [x] All plots are contour plots with outliers or pseudocolor plots.
- [x] A numerical value for number of cells or percentage (with statistics) is provided.

Methodology

Sample preparation	Cells have been collected in eppis after trypsinization from the plate, spin down at 2000rpm for 5min, washed once in PBS, spin down again for 5min at 2000rpm and diluted in 200-500ul PBS for flow cytometry analysis or sorting. Cells were kept on ice until analysis.
Instrument	BD FACSCanto™ II Analyzer, BD FACSAria™ Fusion Sorter
Software	Data were collected using BD Diva software. Data were analyzed using FlowJo Treestar v10
Cell population abundance	When analyzing stable cell lines, all or nearly all cells were positive for the YFP, YFP+tBFP or GFP- tagged reporter constructs. When analyzing transiently transfected fluorescently labeled constructs, the positive population varied based on the transfcetion efficiency and expression levels, mostly between 10-50% positive cells.
Gating strategy	We gated first for living cells based on SSC-A vs FSC-A. We gated then for single cells based on FSC-A vs FSC-W. Then:<br>a) Stable cell lines: We analyzed the median fluorescent intensity directly from all living and single cells.<br>b) Transiently transfected constructs: We gated the positive cells based on a negative sample and analyzed from there the median fluorescent intensity and/or the percentage of positive cells.<br>c) Soluble/aggregated constructs: We gated FITC-H vs FITC-W and selected aggregates based on increased FITC-H and decreased FITC-W and soluble as the population with the higher FITC-W. From there, we analyzed the median fluorescent intensity and/or the percentage of positive cells.

- [x] Tick this box to confirm that a figure exemplifying the gating strategy is provided in the Supplementary Information.
