# Supplementary figures and images for: Mitochondria serve as a holdout compartment for aggregation-prone proteins hindering efficient degradation
Source: Nat Commun. 2026 May 7;17:4195. doi: 10.1038/s41467-026-72783-0 (PMC13153185; doi:10.1038/s41467-026-72783-0)

Figure 1b

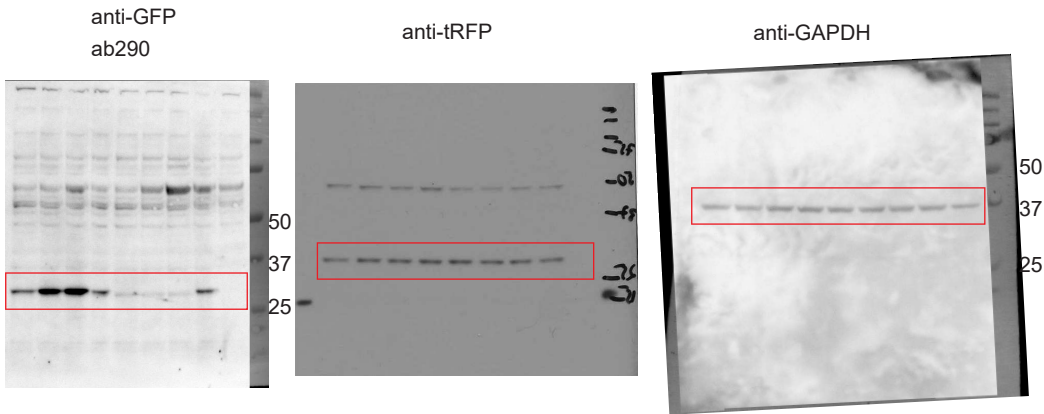

Fig.5e

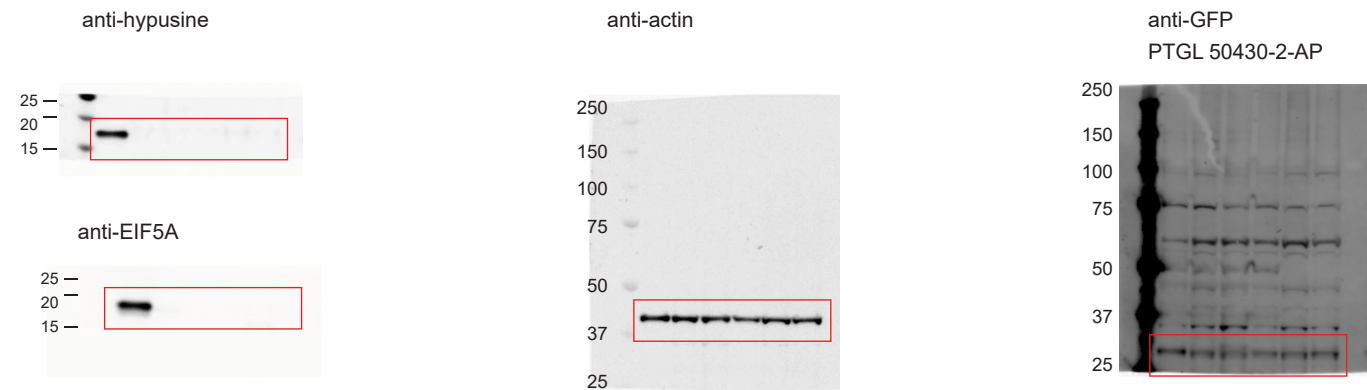

Figure 6b

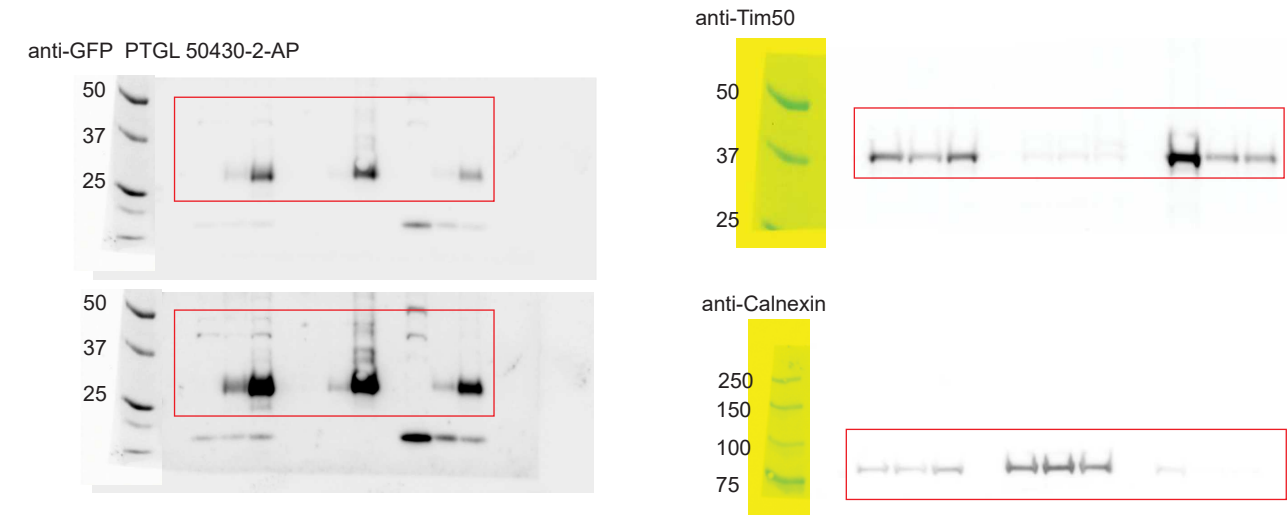

Figure 6c

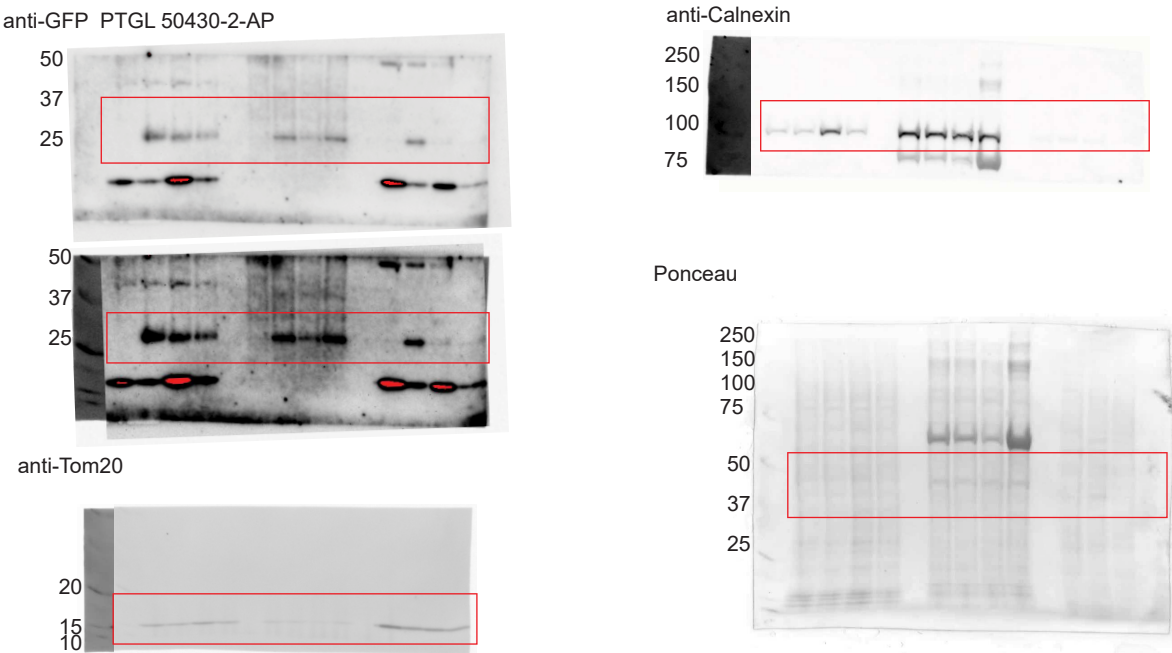

Figure 7b

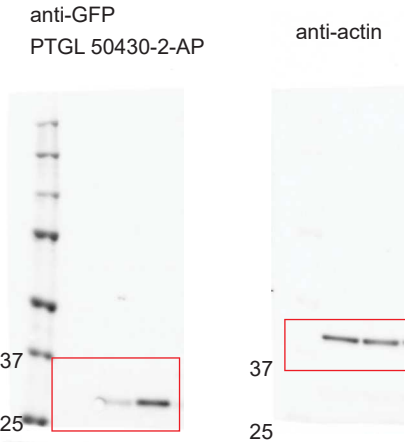

Figure 7d

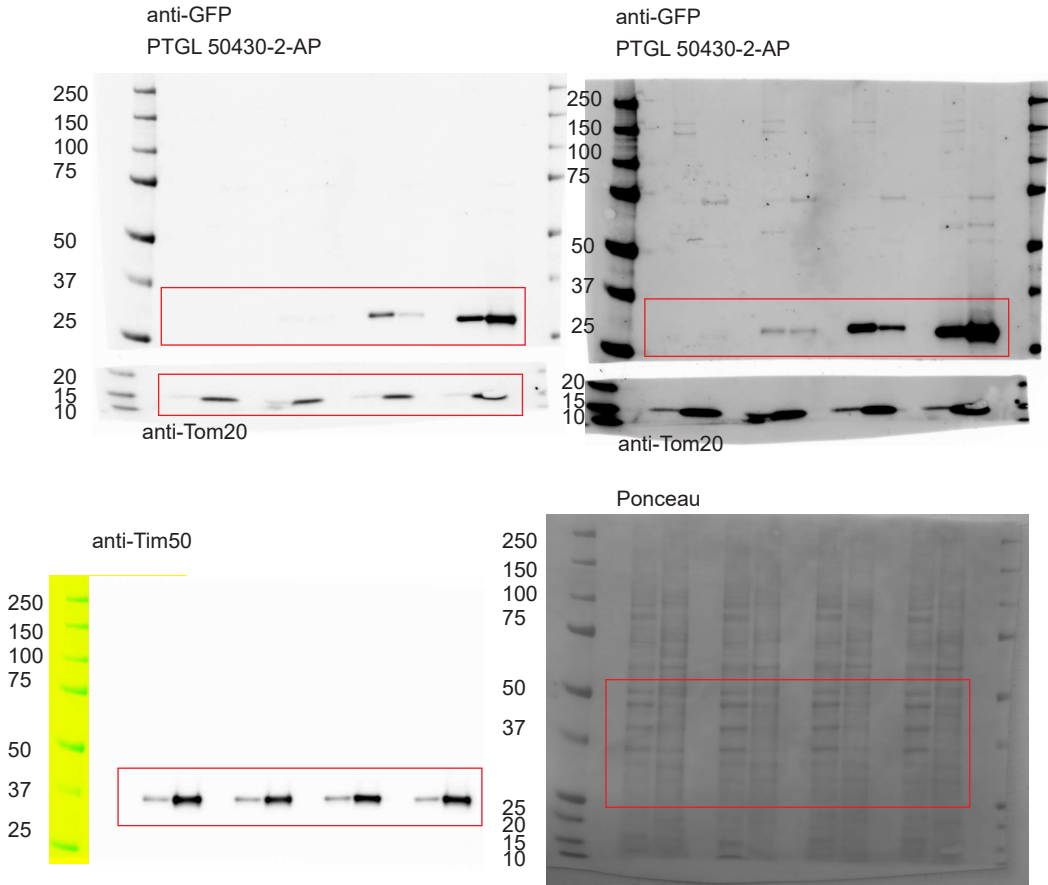

Figure 8f

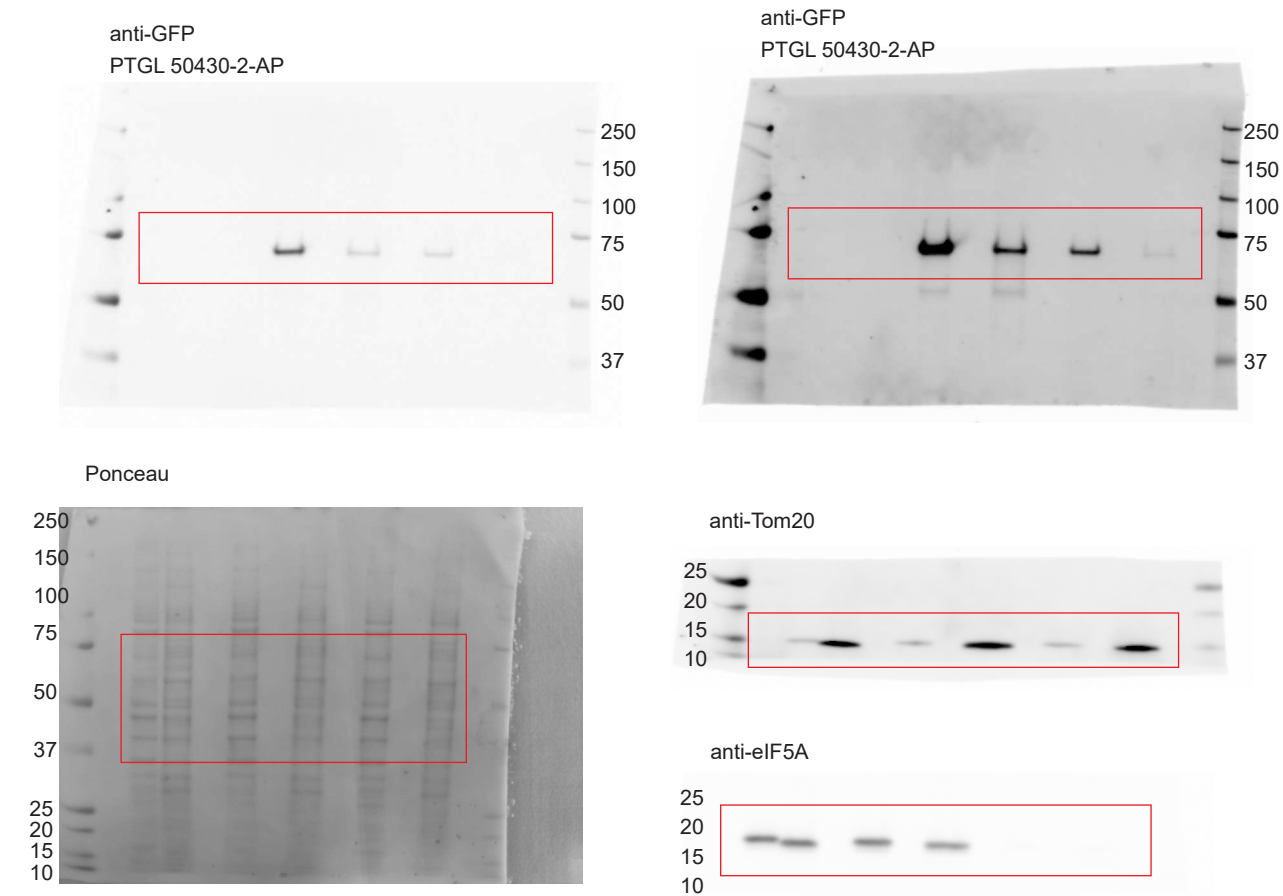

Supplement: Supplementary file 6 — Source Data 1 [file 41467_2026_72783_MOESM6_ESM.zip › Source Data File uncropped blots Main Figures.pdf]
